# Herbicidal Activity and Metabolic Profiling of *Piper tuberculatum* Jacq. Leachates

**DOI:** 10.1021/acs.jafc.4c11286

**Published:** 2025-03-27

**Authors:** Yanka Manoelly dos Santos Gaspar, Álex Ap. Rosini Silva, Andreia M. Porcari, Francisca Diana da Silva Araújo

**Affiliations:** † Postgraduate Program in Agricultural Sciences, Campus Professora Cinobelina Elvas, Federal University of Piauí, Bom Jesus, PI 64900-000, Brazil; ‡ MS4Life Laboratory of Mass Spectrometry, Health Sciences Postgraduate Program, São Francisco University, Bragança Paulista, São Paulo, SP 12916-900, Brazil

**Keywords:** allelopathy, *Bidens bipinnata*, *Digitaria insularis*, molecular networking, natural herbicide

## Abstract

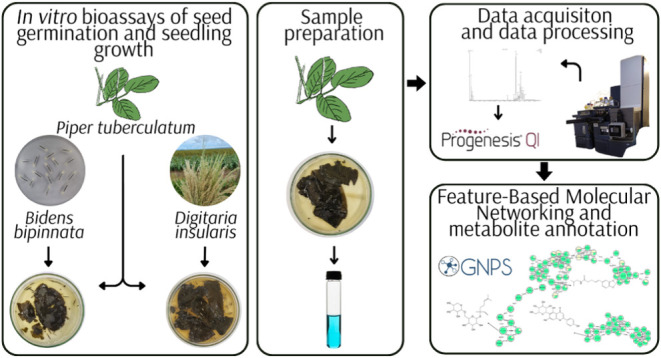

Understanding how allelochemicals with herbicidal activity are released in plant interactions is key to developing sustainable weed control strategies. This study aimed to investigate the herbicidal activity and metabolic profile of *Piper tuberculatum* Jacq. leachates. *In vitro* bioassays were performed with *P*. *tuberculatum* leaf leachates to evaluate their effects on the germination and early growth of *Bidens bipinnata* L. and *Digitaria insularis* (L.) Fedde. (DIGIN.). The leachate extracts were subsequently characterized via liquid chromatography high-resolution mass spectrometry–based metabolomics and molecular networking. The results showed that weed germination and seedling development were significantly affected by the *P*. *tuberculatum* leachates. Metabolomic analysis revealed that allelochemicals belonging to the classes of alkaloids, fatty acids, phenolic compounds, steroids, and terpenoids are potentially involved in herbicidal activity. These findings suggest that *P*. *tuberculatum* could be explored as a natural alternative for sustainable weed management, potentially reducing the dependence on synthetic herbicides.

## 1 Introduction

One of the main factors that restricts food production in agricultural systems is weeds, which grow simultaneously with crops, reducing productivity and product quality.^1^ The constant practice of monoculture and prolonged use of a single herbicide over several decades have played a significant role in the natural selection of resistant or tolerant plant biotypes, in addition to causing harmful effects on human health and the environment.^2^

*Bidens bipinnata* L. is a poorly studied weed with invasive potential in cultivated areas that can have negative consequences on agricultural production.^3,4^ This species belongs to the Asteraceae family and is widely distributed in tropical, subtropical, and temperate regions.^5^ A study on the floristic diversity of invasive weeds in vineyards revealed the occurrence of 14 distinct species, including *B*. *bipinnata*.^6^ There are studies on the competition of this species with the dominant crop^7^ and its role as a host for pests and diseases.^8^

*Digitaria insularis* (L.) Fedde. (DIGIN.) is one of the main weeds that infest South American crops; it is native to the American continent and is common in coffee crops, orchards, and oilseed areas, such as those of soybeans.^9,10^ It is a perennial plant from the Poaceae family that can germinate, grow, and develop practically throughout the year. Its control is difficult because of its ability to form rhizomes.^11^ The seeds have a dense covering of hairs that contribute to their dispersion over considerable distances. This characteristic, combined with its high germination rate, facilitates species dissemination,^9^ making it a highly aggressive invasive plant.

Allelopathic plants have been widely explored as environmentally friendly weed control methods.^12−15^ Allelopathy consists of the stimulating or inhibitory effect that one plant exerts on another through the release of allelochemicals into the environment.^16^ Terpenoids, phenolic compounds, and alkaloids are among the main classes of allelochemicals involved in allelopathy.^17^ They can be released into the environment through four pathways: volatilization; leaching by rain, dew, and fog rain; exudation from roots; and decomposition of plant residues.^18^

Few studies have investigated the allelopathic potential of *Piper tuberculatum* Jacq. in weed control. This species is widely distributed across the American continent and the Antilles^19^ and produces especially amides with several biological activities.^20−23^ Previous reports have demonstrated the phytotoxicity of this species against *Lactuca sativa* L.,^24,25^*Mimosa pudica* L., *Senna obtusifolia* (L.) H. Irwin & Barneby,^26^ and *B*. *bipinnata*.^27^ However, there are no reports on the release pathways of allelochemicals from *P*. *tuberculatum* to the environment.

Therefore, in this study, we investigated the allelochemicals released by *P*. *tuberculatum* through leaching and their herbicidal activity against weeds. *In vitro* bioassays were carried out with leachates from *P*. *tuberculatum* leaves to evaluate the effects on the seed germination and seedling growth of *B*. *bipinnata* and *D*. *insularis*. Leachates were characterized via liquid chromatography–tandem mass spectrometry–based metabolomics and molecular networking.

## 2 Materials and Methods

### 2.1 Plant Material and Chemicals

Leaves from the aerial part of *P*. *tuberculatum* and seeds of *B*. *bipinnata* and *D*. *insularis* were collected in the morning in a native area in the region of Bom Jesus-PI (9°04′26.6″S 44°20′31.1″W, 9°04′56.8″S 44°19′41.8″W, and 9°12′48″S 44°54′48″W, respectively). The exsiccates were deposited in the Graziela Barroso Herbarium (Federal University of Piauí, Teresina, PI, Brazil) under the registration numbers TEPB 32521 (*B*. *bipinnata*), TEPB 32847 (*D*. *insularis*), and TEPB 32850 (*P*. *tuberculatum*). The plants were identified after collection. The seeds were subsequently cleaned manually, and leaves, twigs and other impurities were removed. The plants were registered in the National System for the Management of Genetic Heritage and Associated Traditional Knowledge (SisGen) by n° AE123A5 and A38AE8E.

Liquid chromatography–mass spectrometry (LC–MS)-grade methanol and acetonitrile and analytical grade formic acid (FA) and sodium formate encephalin were purchased from J.T. Baker (Center Valley, PA). Leucine enkephalin was purchased from Waters (Manchester, UK).

### 2.2 *In Vitro* Bioassays of Seed Germination and Seedling Growth

For the leachate bioassay, the sandwich method was used,^28^ where 10 mL of 0.5% agar solution was placed in a Petri dish; subsequently, 25, 50, 100, and 200 mg of dried *P*. *tuberculatum* whole leaves were added, and another 10 mL of agar solution was added. The leaves used were previously dried in an oven with forced air circulation at 45 °C for 72 h. In the control treatment, there was no addition of plant material to the agar. Then, 25 weed seeds were added to each plate. The bioassay was carried out with 16 replications totaling 400 seeds per treatment and was conducted in accordance with a completely randomized design. The 16 replications were grouped into 4 replications of 100 seeds per treatment, as recommended by the Rules for Seed Analysis (RAS).^29^ The plates were kept in a germination chamber under conditions of constant temperature (25 °C for *B*. *bipinnata* and 35 °C for *D*. *insularis*) and controlled light (photoperiod of 12 h) for 7 days. The incubation temperatures of the plant seeds were defined according to the recommendations for germination tests described in the RAS.^29^ After this period, the root and hypocotyl lengths were measured with a digital calliper.

The germination assessment was carried out following the recommendations of the Rules for Seed Analysis;^29^ all those that showed the essential structures of the embryo developed and had a radicle length of at least 2 mm were considered normal. The variables germination percentage (*G*), germination speed index (GSI), and allelopathic effect response index (RI) were analyzed. *G* was calculated via the formula *G* = (N/A) × 100, where *N* represents the number of germinated seeds and *A* represents the total number of seeds. The GSI was determined via the formula GSI = (G1/N1 + G2/N2 + ... + Gn/Nn), where G1, G2, and Gn represent the number of seeds that germinated in the first, second, and last counts, respectively, and N1, N2, and Nn represent the number of days that elapsed until the last count.^30^

The RI was calculated via the formula RI = 1 – *C*/*T* (when *T* ≥ *C*) and RI = *T*/*C* – 1 (when *T* < C), where *C* is the germination speed of the control and *T* is the germination speed of the treatment. A positive RI indicates a stimulatory effect, whereas a negative value indicates an inhibitory effect, with the absolute value being related to the intensity of the allelopathy. The Shapiro–Wilk test was applied to assess data normality, followed by analysis of variance (ANOVA) via SigmaPlot v14.0 software (Systat Software, Inc., Chicago). The criterion for statistical significance was *p* < 0.05.

### 2.3 Characterization of *P*. *tuberculatum* Leachates by Mass Spectrometry-Based Metabolomics

#### 2.3.1 Sample Preparation

Petri dishes containing 0.5% agar solution and 200 mg of previously dried leaves were prepared as previously described and kept in a germination chamber at 25 °C for 7 days. The agar solution containing the leachates was subsequently removed from the Petri dish, extracted with methanol (15 mL), vortexed for 1 min, allowed to rest for 5 min, and stirred again for 1 min. After filtration, the supernatants were concentrated to approximately 1 mL, lyophilized, and stored in a freezer until use. Control samples were obtained via the extraction of 0.5% agar solution (20 mL) without adding the leaves. All the samples were prepared in sextuplicate.

#### 2.3.2 Analysis by Liquid Chromatography High-Resolution Mass Spectrometry (LC–HRMS)

Freeze-dried samples were reconstituted in a solution containing water/methanol/acetonitrile (1:2:2 v/v/v, 1 mL), vortexed for 1 min, sonicated for 30 min at room temperature, and filtered through a 0.22 μm PTFE syringe (Millipore). An ACQUITY UPLC system was used for sample analysis and connected to an XEVO-G2XS QTOF mass spectrometer (Waters, Manchester, United Kingdom) equipped with an electrospray ionization source. Liquid chromatography was performed using a Titan C18 UHPLC column (2.1 × 100 mm, 1.9 μm, Supelco) maintained at 45 °C. The separation was conducted at a flow rate of 0.4 mL min^–1^ following a gradient program in which the mobile phase consisted of (A) 0.1% formic acid (v/v) and (B) pure methanol. The gradient program was applied as follows (in %B): (*t*) = 0 min, 1%; *t* = 2.0 min, 1%; *t* = 8.0 min, 38%; *t* = 20 min, 99.5%; *t* = 25 min, 99.5%; *t* = 25.1 min, 1%; and *t* = 28 min, 1%, resulting in a total analysis time of 28 min. The injection volume was 0.2 μL.

Positive and negative ion modes were recorded separately, and the instrument was operated in data-independent acquisition (MS^E^) mode. The *m*/*z* range varied from 100 to 1700, with an acquisition rate of 0.5 s per scan. The instrument parameters included capillary pressure at 3.0 kV, cone voltage at 40,000 V, desolvation temperature at 550 °C, gas flow in the cone at 10 L h^–1^, and desolvation gas flow at 900 L h^–1^. The collision energy was 20 to 60 eV for fragmentation. Leucine enkephalin (molecular weight = 555.62; 200 pg μL^–1^ in 1:1 acetonitrile:water) was used as the lock mass for accurate mass measurements, and 0.5 mM sodium formate solution was used for calibration. The samples were analyzed randomly.

#### 2.3.3 Data Processing

LC–MS raw data were processed via Progenesis QI 2.0 software (Nonlinear Dynamics, Newcastle, UK), which enables the selection of possible adducts, peak alignment, deconvolution, and putative metabolite identification on the basis of MS^E^ experiments. The Vaniya/Fiehn Natural Products Library,^31^ the MassBank database,^32^ the RIKEN Plant Metabolome MetaDatabase,^33^ the ReSpect Database for Phytochemicals,^34^ HMDB (Human Matabolome Database)^35^ and the LIPID MAPS Database^36^ were used to perform identification via the following search parameters: precursor mass error ≤ 5 ppm and fragment tolerance ≤ 10 ppm. Only mass features with a fragmentation score ≥ 40.0 for each ion mode were annotated.

#### 2.3.4 Feature-Based Molecular Networking

The raw LC–HRMS data were imported into Progenesis QI v2.0 software (Nlinear Dynamics, Newcastle, UK), where peak alignment, peak picking, and deconvolution were performed. The ion quantification tables (.csv) and MS/MS spectral summaries (.msp) were exported and subsequently uploaded to the Global Natural Products Social Molecular Network (GNPS) platform^37^ and analyzed via the feature-based molecular network (FBMN) tool.^38^

The data were filtered by removing all MS/MS fragment ions within ±17 Da of the precursor ion. MS/MS spectra were window filtered by choosing only the top 6 fragment ions in the ±50 Da window throughout the spectrum. The precursor ion mass tolerance and MS/MS fragment ion tolerance were set to 0.02 Da. A molecular network was created in which edges were filtered to obtain a cosine score above 0.7 and more than 4 matched peaks. Edges between two nodes were retained in the network if each of the nodes appeared in the respective top 10 most similar nodes. Finally, the maximum size of a molecular family was set to 100, and the lowest scoring edges were removed from the molecular families until their size was below this threshold.

The spectra in the network were searched in GNPS spectral libraries.^32,37^ The library spectra were filtered in the same way as the input data. The FBMN results on the GNPS platform can be accessed for both positive and negative modes at https://gnps.ucsd.edu/ProteoSAFe/status.jsp?task=be52639315c94d2c8284fab76663f346 and https://gnps.ucsd.edu/ProteoSAFe/status.jsp?task=56e345566f1e4e2b90361b09763a68b6, respectively. Molecular networks were visualized via Cytoscape software version 3.10.1 (Cytoscape Consortium, San Diego, CA).^39^

## 3 Results and Discussion

### 3.1 *P*. *tuberculatum* Leachates Inhibit the Seed Germination and Early Growth of Weeds

**Figure 1:**
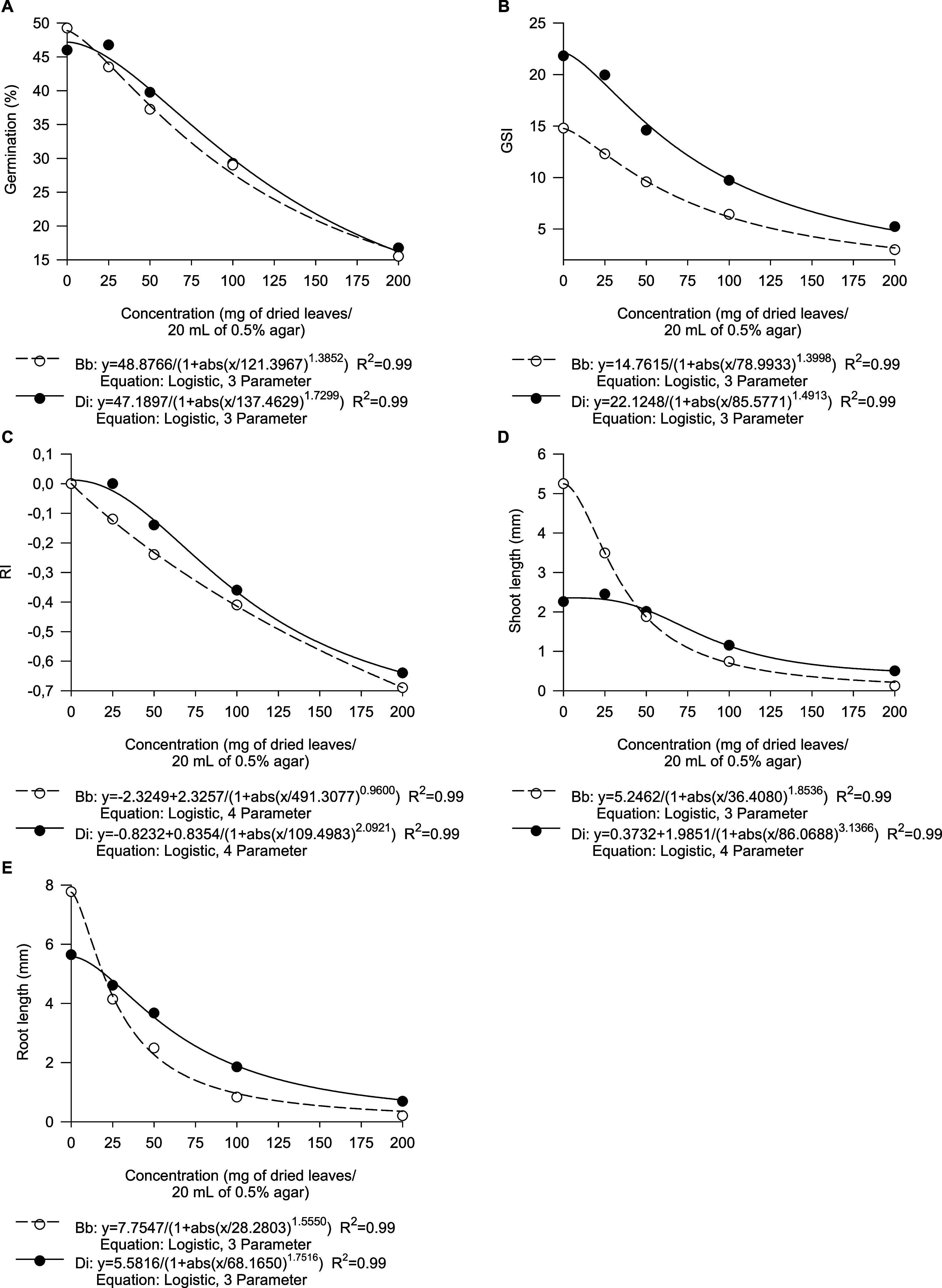
Dose–response curves for the germination percentage (A), germination speed index (GSI) (B), allelopathic effect index (RI) (C), and shoot (D) and root (E) lengths of *B*. *bipinnata* (Bb) and *D*. *insularis* (Di) treated with leachates from *P*. *tuberculatum* leaves.

**Figure 2:**
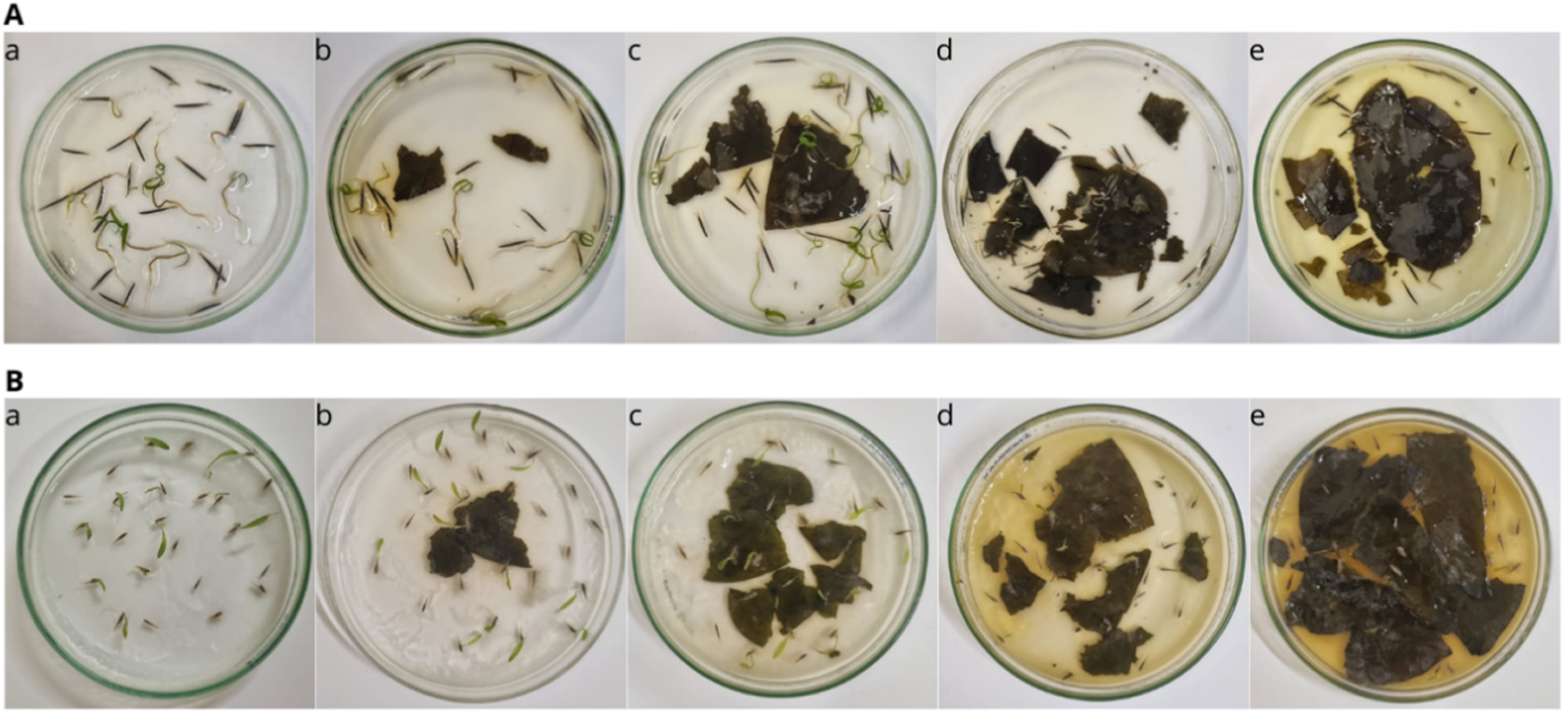
Inhibition of seed germination and seedling growth of the weeds *B*. *bipinnata* (A) and *D*. *insularis* (B) by *P*. *tuberculatum* leachates using 0 (a), 25 (b), 50 (c), 100 (d), and 200 mg (e) of dried leaves/20 mL of 0.5% agar solution.

The seed germination and seedling growth of *B*. *bipinnata* and *D*. *insularis* were investigated after treatment with leachates from *P*. *tuberculatum* leaves, aiming to increase the understanding of the allelopathy of this plant for use as a natural herbicide. There was a reduction in the germination percentage, germination speed index, allelopathic effect response index, and length of shoot and root of the weeds proportional to the increase in *P*. *tuberculatum* leaf biomass used in the bioassay (Tables S1 and S2), with significant differences between the different doses (*p* < 0.001) (Tables S3 and S4). Dose–response curves for all the parameters were fitted to a logistic regression model for *B*. *bipinnata* and *D*. *insularis* (Figure 1), and the strongest inhibition was achieved with 200 mg of dried leaves/20 mL of 0.5% agar (Figure 2).

Our results demonstrated that *P*. *tuberculatum* uses the leaching pathway for communication with other plants. This species probably also uses volatilization, decomposition, and root exudation pathways to release allelochemicals into the environment; however, this work focused on specifically demonstrating the use of the leaching route. Once carried by leaching, these compounds must reach neighboring plants and act to inhibit them, aiming at the prevalence of the species in their habitat. These compounds can cause phytotoxicity, harm plant development or even affect the germination of seeds of different plant species present in the soil close to *P*. *tuberculatum*. Additionally, allelochemicals released by leaching can also be deposited and concentrated in the soil and, over time, acquire toxic effects.^40^ Studies involving the structural modifications of these allelochemicals when released into the environment are crucial for obtaining a complete understanding of their inhibition mechanisms. They can undergo structural changes, for example, by the soil microbiota.^17^

Importantly, studies that prove the involvement of a certain molecule in allelopathy are challenging, as it is necessary to use methodologies that simulate natural conditions and prove that these compounds are released by the plant.^41,42^ This occurs because many phytotoxic compounds produced by plants are not released by them, and some of these substances may even lose their phytotoxicity when released into the environment.^43^ The allelochemicals involved in allelopathy are released by plants and act by inhibiting or stimulating the germination and development of other plants.^16^ In other words, not all phytotoxic compounds produced by plants are allelopathic, although the literature is full of studies that equate the phytotoxicity of a compound found in a plant with allelopathy.^44^ These peculiar characteristics of allelopathic compounds make them promising for application in the agricultural sector, whether for weed inhibition or as growth stimulators for cultivated plants.

In this sense, the sandwich method used in our study is useful for screening the allelopathic effects of plant leachates under laboratory conditions, and its application is highly relevant for replicating natural conditions.^45^ By transferring water-soluble allelochemicals from leaves to a substrate such as agar, this method effectively simulates the leaching of compounds, an essential process in ecosystem dynamics. This approach allows for an efficient assessment of allelopathic effects, in addition to significantly reflecting the complex interaction between plants and the environment, contributing to a deeper understanding of the underlying ecological processes.^45^ The sandwich method is a less time-consuming and easier-to-perform bioassay, making it valuable for screening large numbers of samples. In allelopathy studies, where it is common to test multiple plant species and different chemical compounds, the ability to process many samples efficiently is important to advance the understanding of allelopathic effects. More than 852 plant species have already been screened via this methodology.^28,45−48^

To our knowledge, this is the first study demonstrating the release of allelochemicals by leaching from *P*. *tuberculatum*, in addition to being a pioneer in investigating the impact of these leachates on the inhibition of the weeds *B*. *bipinnata* and *D*. *insularis*. These allelochemicals likely also have inhibitory effects on other weeds; therefore, additional studies with different species are needed to confirm this hypothesis. The validation of the allelopathic and herbicidal effects of the allelochemicals present in *P*. *tuberculatum* leachates on other weeds opens a range of opportunities for their application in integrated weed management. Therefore, it is crucial to identify the structures of these compounds and prove their individual and synergistic effects.

### 3.2 Metabolic Profiling of *P*. *tuberculatum* Leachates

A total of 41 compounds belonging to the classes of alkaloids, carboxylic acids, phenolic compounds, and terpenoids were annotated in the *P*. *tuberculatum* leachates. The precursor ion of *m*/*z* 158.0819 [M-H]^−^ was annotated as betonicine (**1**) (Table 1), an alkaloid previously reported in seeds of *Trigonella caerulea* (L.) Ser.^49^ Alkaloids are extensively distributed throughout the plant kingdom and often exert significant defensive effects on plants via interactions with herbivores, microorganisms, fungi, and neighboring plants.

**Table 1:** Secondary Metabolites Annotated in *P*. *tuberculatum* Leachates via UPLC-ESI-QTOF-MS

no.	retention time (min)	precursor ion	adduct type	molecular formula	exact mass	compound	Δ*m*/*z* (ppm)
Alkaloid							
1	4.89	158.0819	[M-H]^−^	C_7_H_13_NO_3_	159.0895	betonicine	–2.29
Carboxylic Acid							
2	0.89	237.0613	[M + FA-H]^−^	C_7_H_12_O_6_	192.0634	quinic acid	–1.09
3	6.60	147.0294	[M-H]^−^	C_5_H_8_O_5_	148.0372	citramalic acid	–3.19
Phenolic Compounds							
4	0.67	395.1131	[M-H_2_O–H]^−^	C_22_H_22_O_8_	414.1315	6,4′-dihydroxy-7-methylaurone 6-*O*-deoxyhexoside	–1.18
5	4.54	814.2328	[M + FA-H]^−^	C_38_H_41_O_17_^+^	769.2338	peonidin 3-*O*-hexoside-coumaroyl 5-*O*-hexoside	0.39
6	7.07	511.1783	[M-H_2_O–H]^−^	C_31_H_30_O_8_	530.1941	artonin Q	4.01
7	7.20	611.1973	[M-H]^−^	C_28_H_36_O_15_	612.2054	neohesperidin dihydrochalcone	–1.28
8	7.96	755.2040	[M-H]^−^	C_33_H_40_O_20_	756.2113	apigenin 8-*C*-hexoside-hexoside-hexoside	0.02
9	8.63	639.1920	[M + H]^+^	C_29_H_34_O_16_	638.1847	rhamnazin 3-*O*-hexoside- deoxyhexoside	0.13
10	8.63	705.1671	[M-H_2_O–H]^−^	C_32_H_36_O_19_	724.1851	isorhamnetin 3-*O*-hexoside-diacetyl-hexoside	–0.24
11	8.64	485.1085	[M-H_2_O–H]^−^	C_24_H_24_O_12_	504.1268	chryseriol 8-*C*-acetyl-hexoside	–0.82
12	8.74	649.1404	[M-H_2_O–H]^−^	C_29_H_32_O_18_	668.1589	dillenetin 5-*O*-hexoside 7-*O*-glucuronide	–0.86
13	9.03	605.1509	[M-H_2_O–H]^−^	C_28_H_32_O_16_	624.1690	3,4′-*O*-dimethyl quercetin 7-*O*-hexoside-pentoside	–0.32
14	9.05	723.1752	[M-H_2_O–H]^−^	C_32_H_38_O_20_	742.1956	quercetin 3-*O*-hexoside-deoxyhexoside-pentoside	–3.50
15	9.12	381.0620	[M-H_2_O–H]^−^	C_20_H_16_O_9_	400.0794	3,5,7,4′-tetrahydroxy-8-C-(3-methylsuccinoyl)flavone	1.06
16	9.12	631.1250	[M + Na]^+^	C_27_H_28_O_16_	608.1377	apigenin 7-*O*-hexoside-glucuronide	–3.26
17	9.12	287.0544	[M + H]^+^	C_15_H_10_O_6_	286.0477	kaempferol	–1.93
18	9.14	755.2039	[M-H]^−^	C_33_H_40_O_20_	756.2113	kaempferol 3-*O*-hexoside-hexoside-deoxyhexoside	–0.16
19	9.16	917.2584	[M-H]^−^	C_39_H_50_O_25_	918.2641	quercetin 3-*O*-deoxyhexoside-hexoside 7-*O*-hexoside- deoxyhexoside	1.70
20	9.16	489.1033	[M-H_2_O–H]^−^	C_23_H_24_O_13_	508.1217	limocitrin 3-*O*-hexoside	–1.12
21	9.16	429.0816	[M-H_2_O–H]^−^	C_21_H_20_O_11_	448.1006	carthamone	–2.45
22	9.18	577.1208	[M + FA-H]^−^	C_25_H_24_O_13_	532.1217	maritimetin 6-*O*-diacetyl-hexoside	1.77
23	9.19	579.1353	[M-H]^−^	C_26_H_28_O_15_	580.1428	kaempferol 3-*O*-pentoside-hexoside	–0.39
24	9.33	755.2044	[M + FA-H]^−^	C_32_H_38_O_18_	710.2058	kaempferol 3-*O*-deoxyhexoside-pentoside 7-*O*-deoxyhexoside	0.51
25	9.48	912.2092	[M + Cl]^−^	C_40_H_45_O_22_^+^	877.2397	pelargonidin 3-*O*-hexoside 7-*O*-hexoside-*p*-hydroxybenzoyl-hexoside	–0.49
26	9.49	499.0857	[M-H_2_O–H]^−^	C_24_H_22_O_13_	518.1060	apigenin 7-*O*-hexoside-malonyl	–4.80
27	9.51	282.0523	[M-H_2_O–H]^−^	C_16_H_14_O_6_	302.0790	2,5,7-trihydroxy-4′-methoxyisoflavanone	–3.49
28	9.92	679.1519	[M-H]^−^	C_30_H_32_O_8_	680.1589	chryseriol 7-*O*-hexoside-malonyl-pentoside	0.46
29	10.09	605.1497	[M-H_2_O–H]^−^	C_28_H_32_O_16_	624.1690	syringetin 3-*O*-pentoside- deoxyhexoside	–2.30
30	10.33	713.1715	[M + FA-H]^−^	C_33_H_32_O_15_	668.1741	4-*O*-methyl okanin 4′-*O*-acetyl-hexoside-caffeoyl	–1.18
31	10.49	323.0556	[M-H_2_O–H]^−^	C_18_H_14_O_7_	342.0740	apigenin 7-*O*-lactate	–1.45
32	10.49	443.0970	[M-H_2_O–H]^−^	C_22_H_22_O_11_	462.1162	6-*C*-methylkaempferol 3-*O*-hexoside	–2.91
33	10.73	606.1241	[M-H_2_O–H]^−^	C_27_H_29_O_17_^+^	625.1399	cyanidin 3-*O*-hexoside-glucuronide	2.40
34	10.99	753.2022	[M + H–H_2_O]^+^	C_37_H_38_O_18_	770.2058	isorhamnetin 3-*O*-hexoside-deoxyhexoside-coumaroyl	–0.45
35	11.11	325.0902	[M + H–H_2_O]^+^	C_15_H_18_O_9_	342.0951	3-(4-hydroxyphenyl)propanoic acid 3-*O*-glucuronide	–4.49
36	11.11	641.1525	[M + H]^+^	C_31_H_28_O_15_	640.1428	quercetin 3-*O*-hexoside-feruloyl	3.78
Terpenoids							
37	4.80	401.1091	[M + FA-H]^−^	C_16_H_20_O_9_	356.1107	gentiopicroside	0.64
38	9.49	543.1463	[M + Na]^+^	C_25_H_28_O_12_	520.1581	2′-*O*-coumaroylgardoside	–1.78
39	9.65	509.2234	[M + FA-H]^−^	C_21_H_36_O_11_	464.2258	linalool oxide D 3-*O*-hexoside-pentoside	–1.20
40	11.11	193.0865	[M + H]^+^	C_11_H_12_O_3_	192.0786	shinanolone	3.09
41	12.72	358.1276	[M + H–H_2_O]^+^	C_16_H_23_O_10_^–^	375.1297	loganate	4.83

The precursor ions of *m*/*z* 237.0613 [M + FA-H]^−^ and 147.0294 [M-H]^−^ were annotated as quinic (**2**) and citramalic (**3**) acids, respectively (Table 1). Quinic acid, which is also found in the aerial parts and roots of *Hypericum empetrifolium* Willd.,^50^ has several biological properties, including antibacterial, antioxidant, and antiviral properties.^51−53^ Citramalic acid is a dicarboxylic fatty acid also found in beet root exudates and has the ability to solubilize phosphorus in the soil, increasing its availability.^54^ Fatty acids play a crucial role in allelopathy because of their inhibitory properties,^55,56^ which can alter the permeability of the cell membranes of target organisms, resulting in leakage of cellular contents, damage to membrane structures, and impaired toxin synthesis;^57^ they can also reduce the chlorophyll content and cause oxidative stress.^58^

Phenolic compounds, including 33 compounds (**4**-**36**) from the subclasses of aurones, glycosylated flavonoids, flavonols, phenolic acids, flavonones, anthocyanidins, flavones, iridoid glycosides, coumarins, and isoflavonoids (Table 1), were the most common in the *P*. *tuberculatum* leachate. In the aurona subclass, the compounds 6,4′-dihydroxy-7-methylaurone 6-*O*-deoxyhexoside (**4**) and maritimetin 6-*O*-diacetyl-hexoside (**22**) were annotated, with precursor ions of *m*/*z* 395.1131 [M-H_2_O–H]^−^ and 577.121 [M + FA-H]^−^, respectively, and were previously isolated from *Pterocarpus marsupium* Roxb^59^ and *B*. *bipinnata*.^60^

Several compounds belonging to the flavonol subclass have been identified. Among them, compound **18**, with a precursor ion of *m*/*z* 287.054 [M + H]^+^, was annotated as kaempferol and its derivatives with precursor ions of *m*/*z* 755.204 [M-H]^−^, 579.135 [M-H]^−^, 755.204 [M + FA-H]^−^, and 443.097 [M-H_2_O–H]^−^, annotated as kaempferol 3-*O*-hexoside-hexoside-deoxyhexoside (**18**), kaempferol 3-*O*-pentoside-hexoside (**23**), kaempferol 3-*O*-deoxyhexoside-pentoside 7-*O*-deoxyhexoside (**24**), and 6-*C*-methylkaempferol 3-*O*-hexoside (**32**), respectively. Kaempferol is a type of flavonol with antioxidant properties that is widely distributed in citrus fruits, vegetables, and herbs.^61,62^

The compounds **6**, **9**, **10**, **20**, **29**, and **34** were annotated as artonin Q (*m*/*z* 511.178 [M-H_2_O–H]^−^), rhamnazin 3-*O*-hexoside-deoxyhexoside (*m*/*z* 639.192 [M + H]^+^), isorhamnetin 3-*O*-hexoside-diacetyl-hexoside (*m*/*z* 705.167 [M-H_2_O–H]^−^), limocitrin 3-*O*-hexoside (*m*/*z* 489.103 [M-H_2_O–H]^−^), syringetin 3-*O*-pentoside-deoxyhexoside (*m*/*z* 605.15 [M-H_2_O–H]^−^), and isorhamnetin 3-*O*-hexoside-deoxyhexoside-coumaroyl (*m*/*z* 753.202 [M + H–H_2_O]^+^), respectively. Compounds **6**, **9**, and **20** were previously found in *Artocarpus heterophyllus* Lam.,^63^*Cucurbita pepo* L.^64^ and *Erica cinerea* L.,^65,66^ respectively.

Quercetin derivatives with precursor ions of *m*/*z* 605.1509 [M-H_2_O–H]^−^, 723.175 [M-H_2_O–H]^−^, 917.258 [M-H]^−^, and 641.153 [M + H]^+^ were annotated as quercetin 3,4′-*O*-dimethyl quercetin 7-*O*-hexoside-pentoside (**13**), quercetin 3-*O*-hexoside-deoxyhexoside-pentoside (**14**), quercetin 3-*O*-deoxyhexoside-hexoside 7-*O*-hexoside-deoxyhexoside (**19**), and quercetin 3-*O*-hexoside-feruloyl (**36**), respectively. Quercetin is an inhibitor of auxin, a type of plant hormone essential for plant growth and development, and functions as a protein kinase inhibitor, which can affect cell growth and morphogenesis in plants, as well as inhibiting ATPase, an enzyme that plays a crucial role in cellular energy production by converting ATP to ADP. Another important action of quercetin is the inhibition of electron transport, which can lead to the production of reactive oxygen species (ROS).^67−70^

The flavone subclass included chryseriol 8-*C*-acetyl-hexoside (**11**, *m*/*z* 485.1085 [M-H_2_O–H]^−^), 3,5,7,4′-tetrahydroxy-8-C-(3-methylsuccinoyl)flavone (**15**, *m*/*z* 381.062 [M-H_2_O–H]^−^), and chryseriol 7-*O*-hexoside-malonyl-pentoside (**28**, *m*/*z* 679.152 [M-H]^−^). Compound **28** is a derivative of chryseriol with antibacterial, antifungal, and insecticidal activities^71^ and was previously found in the leaves and stems of *Cydonia oblonga* Mill.^72^ Among the isoflavonoids, the precursor ion at *m*/*z* 282.052 [M-H_2_O–H]^−^ was annotated as 2,5,7-trihydroxy-4′-methoxyisoflavanone (**27**).

The precursor ions of *m*/*z* 814.233 [M + FA-H]^−^ and 912.209 [M + Cl]^−^ were annotated as peonidin 3-*O*-hexoside-coumaroyl 5-*O*-hexoside (**5**) and pelargonidin 3-*O*- hexoside 7-*O*-hexoside-*p*-hydroxybenzoyl-hexoside (**25**) (Table 1), which are derivatives of peonidin and pelargonidin of the anthocyanin and anthocyanidin subclasses, respectively. Like other flavonoids, anthocyanins play several roles in plant defense against other organisms, including chemical repellents and visual signaling,^73^ and some anthocyanins exhibit antiviral, antibacterial, and fungicidal activities.^74^ The precursor ion of *m*/*z* 606.124 [M-H_2_O–H]^−^ annotated as cyanidin 3-*O*-hexoside-glucuronide (**33**) is a derivative of cyanidin, which is also an anthocyanin.

Among the chalcone subclasses, compounds **7**, **21**, and **30** were annotated as neohesperidin dihydrochalcone (*m*/*z* 611.197 [M-H]^−^), carthamone (*m*/*z* 429.082 [M-H_2_O–H]^−^), and 4-*O*-methyl okanin 4′-*O*-acetyl-hexoside-caffeoyl (*m*/*z* 713.172 [M + FA-H]^−^), respectively. The precursor of *m*/*z* 325.09 [M + H–H_2_O]^+^ was annotated as 3-(4-hydroxyphenyl)propanoic acid 3-*O*-glucuronide (**35**), a phenolic acid. In the subclass of iridoid glycosides, compound **8**, with a precursor ion of *m*/*z* 755.2040 [M-H]^−^, was annotated as apigenin 8-*C*-hexoside-hexoside-hexoside (marginatoside), a metabolite found in several plants, including species of the genus *Piper*.^75^

The precursor ions of *m*/*z* 631.125 [M + Na]^+^, *m*/*z* 499.086 [M-H_2_O–H]^−^ and *m*/*z* 323.056 [M-H_2_O–H]^−^ were annotated as apigenin 7-*O*-hexoside-glucuronide (**16**), apigenin 7-*O*-hexoside-malonyl (**26**), and apigenin 7-*O*-lactate (**31**) (Table 1), which are derivatives of apigenin. Apigenin is a flavonoid with phytotoxic activity that affects the germination of *Arabidopsis thaliana* (L.) Heynh. seeds and the growth of the aerial parts and roots of *L*. *sativa*.^76,77^ The precursor ion of *m*/*z* 649.140 [M-H_2_O–H]^−^ annotated as dillenetin 5-*O*-hexoside 7-*O*-glucuronide (**12**) is a derivative of dilenetin, an inhibitor of xanthine oxidase that catalyzes the oxidation of xanthine to uric acid and superoxide, a pathway for O_2_ formation, contributing to the formation of ROS.^78^

Compounds **37**-**41** were found to belong to the terpenoid class (Table 1). The precursor ion of *m*/*z* 401.1091 [M + FA-H]^−^ was annotated as gentiopicroside (**37**), a terpenoid with allelopathic inhibitory activity on the germination and development of *Medicago sativa* L. and *Trifolium pratense* L.^79^ The precursor ions of *m*/*z* 543.1463 [M + Na]^+^, 509.2234 [M + FA-H]^−^, 193.0865 [M + H]^+^, and 358.12761 [M + H–H_2_O]^+^ were annotated as 2′-*O*-coumaroylgardoside (**38**), linalool oxide D 3-*O*-hexoside-pentoside (**39**), shinanolone (**40**), and loganate (**41**), respectively.

Additionally, the metabolite annotation of *P*. *tuberculatum* leachates was also performed via automated library spectral matching with GNPS public spectral libraries, which revealed matches for 10 of 804 and 10 of 376 spectral nodes in the positive and negative modes, respectively (Figure S2). These matches were manually evaluated and compared to the literature, resulting in level 2 annotations according to the Metabolomics Standards Initiative (MSI).^80^ Thus, a total of 14 compounds were annotated, including alkaloids, fatty acids, phenolic compounds, steroids, and terpenoids (Tables S5 and S6).

**Figure 3:**
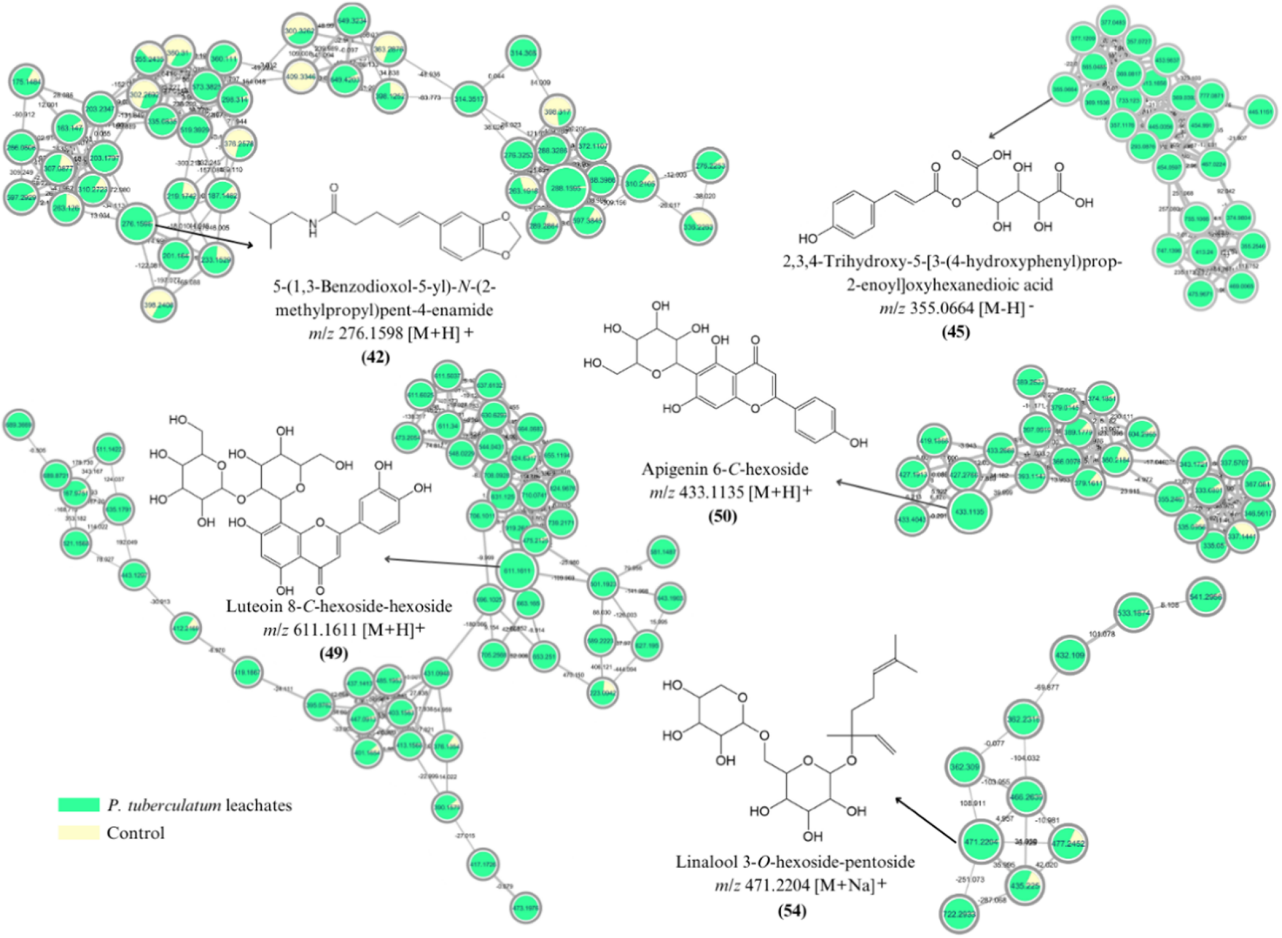
Molecular networks of allelochemicals representative of the classes of alkaloids (**42**), phenolic compounds (**45**, **49**, and **50**), and terpenoids (**54**) generated from the feature-based molecular network (FBMN) workflow and annotated on the basis of spectral matches on the GNPS platform. Each node represents a tandem mass spectrometry (MS/MS) spectrum, while the edges connecting them represent MS/MS fragmentation similarity. These structures are representative, and isomers are possible.

The precursor ion of *m*/*z* 276.1598 [M + H]^+^ was annotated as 5-(1,3-benzodioxol-5-yl)-*N*-(2-methylpropyl)pent-4-enamide **(42)** (Tables S5–S6 and Figure 3), an alkaloid derived from piperlonguminine. Piperlonguminine has already been isolated from *P*. *tuberculatum* roots and has demonstrated larvicidal activity against *Aedes aegypti*.^81^ This compound was also previously isolated from *P*. *retrofractum* and described as phytotoxic, affecting the germination and shoot and root growth of watercress (*Lepidium sativum* L.) and barnyard grass (*Echinochloa colonum* L.).^82^ Piperlonguminine has a methylenedioxyphenyl moiety similar to that of piperine, pipernonaline, or piptigrine. This pharmacophore group plays a significant role in several synergistic agents, such as piperonyl butoxide and its analogues, providing larvicidal, antifeedant, and insecticidal activities.^83−85^

Alkaloids can inhibit plant growth through several mechanisms, such as DNA interference, enzymatic activity, protein biosynthesis, and membrane integrity.^86^ The phytotoxic mode of action of alkaloids generally occurs in two steps. First, these compounds can cross the plasma membrane of plant cells, a process that occurs both in the presence and absence of light. In the second stage, peroxidative activity triggered by singlet oxygen occurs, resulting in reactions that lead to cellular necrosis.^87^ However, the primary targets of cytotoxic alkaloids are DNA, RNA, and several associated enzymes, including telomerases, polymerases, and newly isolated alkaloids, which compromise the stability of unwound DNA chains.^14^

The precursor ion of *m*/*z* 327.2171 [M-H]^−^ was annotated as 9,12,13-trihydroxy-octadec-10,15-dienoic acid **(43)** (Tables S5–S6), which belongs to the fatty acid class. Polyhydroxylated fatty acids, such as 9,12,13-trihydroxy-octadec-10,15-dienoic, 9,12,13-trihydroxy-octadec-10-enoic, and 9,10,13-trihydroxy-octadec-11-enoic acids, which are present in the dichloromethane fraction of vinasse extract, may play a role in phytotoxicity, affecting approximately 72.6% of *L*. *sativa* root growth.^88^ Furthermore, Inderjit and Duke^89^ reported that saturated, unsaturated, and polyunsaturated fatty acids are allelopathic compounds. There is evidence suggesting that fatty acids can influence the cell plasma membrane, mainly by affecting its permeability. This can occur through the formation of an ion channel, leading to disorganization of the membrane structure^90^ and the dissociation of pigments involved in photosynthesis processes.^91^

Among the phenolic compounds, representatives from the subclasses of hydroxycinnamic acid, coumarins, and glycosylated flavonoids were annotated. The precursor ions of *m*/*z* 339.1070 [M + H]^+^, 355.0664 [M-H]^−^, 325.0922 [M-H]^−^, and 177.0550 [M + H–H_2_O]^+^ were annotated as 4-p-coumaroylquinic acid (**44**), 2,3,4-trihydroxy-5-[3-(4-hydroxyphenyl)prop-2-enoyl]oxyhexanedioic acid (**45**) (Figure 3), coumaric acid 4-*O*-hexoside (**47**), and ferulic acid (**48**) (Table S6), respectively, belonging to the class of hydroxycinnamic acids. Ferulic acid has been shown to inhibit the germination and growth of plants of several species, including weeds, by reducing the water content in leaves, affecting root expansion and elongation, compromising photosynthesis, and inhibiting nutrient absorption.^92,93^ 2,3,4-Trihydroxy-5-[3-(4-hydroxyphenyl)prop-2-enoyl]oxyhexanedioic acid is a derivative of coumaric acid and is related to a reduction in the dry weight of plants, chlorophyll content in leaves, osmotic potential, and cell turgor pressure.^94^

The precursor ion of *m*/*z* 315.0716 [M-H]^−^ was annotated as gentisic acid 5-*O*-hexoside (**46**) (Table S6), a diphenolic compound derived from benzoic acid. Benzoic acid has a direct influence on the absorption of ions and minerals, can inhibit the absorption of phosphate (PO_4_^3–^) and potassium (K^+^) ions by barley roots,^95^ and can cause depolarization of the oat coleoptile cell membrane.^96^ However, the benzoic acid present in soil is readily degraded by microbial or chemical processes, meaning that it would need to accumulate at relatively high concentrations in soil, as it is largely absorbed by colloids at relatively low concentrations. Consequently, soil properties play a crucial role in the allelopathic activity of benzoic acid.^97^

The precursor ions of *m*/*z* 611.1611 [M + H]^+^, 433.1135 [M + H]^+^, and 593.1511 [M-H]^−^ were annotated as luteoin 8-*C*-hexoside-hexoside (**49**) (Figure 3), apigenin 6-*C*-hexoside (**50**), and apigenin 8-*C*-hexoside-hexoside (**51**) (Tables S5–S6), respectively, which are classified as glycosylated flavonoids. Glycosylated flavonoids are released through root exudation, tissue degradation or leaching and are found in soil solutions and root exudates.^98^ Apigenin 6-*C*-hexoside has already been reported in the hydroalcoholic extract of *Machaerium eriocarpum* Benth. leaves; this compound exhibits allelopathic inhibitory effects by influencing the germination and growth of sorghum, in addition to inhibiting the formation of lateral roots on cucumber plants.^99^ Luteoin 8-*C*-hexoside-hexoside, previously identified in *Trollius chinensis* Bunge plants, has antibacterial^100^ and antioxidant properties.^101^

The precursor ion of *m*/*z* 316.1909 [M + NH_4_]^+^ was annotated as 7-hydroxy-3-(1,1-dimethylprop-2-enyl)coumarin (**52**) from the hydroxycoumarin class. Coumarins are known for their extensive variety of biological functions and applications as biocides.^102,103^ Most natural coumarins have an oxygenated substituent at the C7 position, with 7-hydroxycoumarin (umbelliferone), 6,7-hydroxycoumarin (esculetin), and 7-hydroxy-6-methoxycoumarin (scopoletin) being the most prevalent in nature.^103,104^ Owing to the possibility that the hydroxyl group at C7 contributes to the phytotoxicity of 7-hydroxycoumarins,^105^ these coumarins have received attention for their possible role in plant–plant allelopathic interactions and their potential as environmentally friendly herbicides.^106−108^

The precursor ion of *m*/*z* 307.2267 [M + H]^+^ was annotated as 17-epioxandrolone (**53**) (Table S6), which belongs to the class of steroids and has already been demonstrated to have an allelopathic effect on *Euphorbia heterophylla*.^17,109^ The precursor ion of *m*/*z* 395.2041 [M + Na-2H]^−^, annotated as tsangane 3-*O*-hexoside (**55**) (Table S6), is a glycosylated terpenoid that functions as a signaling molecule, photoprotective agent, reproductive hormone and allelochemical, contributing to the establishment of plants in the environment.^16,17^ The precursor ion of *m*/*z* 471.2204 [M + Na]^+^, annotated as linalool 3-*O*-hexoside-pentoside (**54**) (Table S6 and Figure 3), is a diglycosylated monoterpene. Several compounds in the form of glycosides are potentially toxic substances that, when combined with sugars, become harmless within the plant.^110^

Among the plant allelochemicals present in the ecosystem, phenolic compounds stand out not only for their physiological relevance but also for their allelopathic potential, interference with multiple enzymes and plant metabolic processes.^111^ Specifically, phenolic acids play a central role as allelochemicals, exerting a significant effect on sensitive species and reducing hydraulic conductivity and net nutrient absorption by roots, thus impacting growth.^112^ They also act by increasing the activity of oxidative enzymes, which results in the modification of membrane permeability and the formation of lignin, thus contributing to the reduction in root growth.^113,114^ A variety of phenolic compounds exert negative allelopathic effects on plant growth by binding to gibberellic acid, inhibiting its activity, whereas others bind to abscisic acid, stimulating growth.^115^ Furthermore, polyphenols such as chlorogenic and isochlorogenic acids, as well as scopoletin coumarin, can neutralize the oxidation of indoleacetic acid.^16^

Terpenoids perform diverse biological functions in plants by acting as hormones, photosynthetic pigments, electron transporters, structural components of membranes and mediators in the assembly of polysaccharides. Furthermore, they actively participate in plant communication and defense.^116^ Most plants produce and release a wide variety of terpenes that, owing to their volatility, help attract pollinators, play a role in defending plants against pathogens and herbivores, and have inhibitory effects on the germination of other plants.^117,118^ Owing to their allelopathic activity, these compounds are considered potential candidates for the development of new agrochemicals based on natural products.^14,119^

Phenolic compounds and terpenoids were previously detected in the aqueous extract of *P*. *tuberculatum* leaves.^27^ Amides and cinnamoyl derivatives were isolated from the seeds and leaves of *P*. *tuberculatum* and shown to have antifungal properties.^20,21^ Cinnamic acid derivatives with repellent effects on leafcutter ants, including piplaroxide, piplartine, and demethoxypiplartine, have also been isolated from *P*. *tuberculatum* leaves.^120^ Studies have demonstrated the antifungal and herbicidal activities of the essential oil from *P*. *tuberculatum* leaves, which is composed mainly of sesquiterpenes.^21,25,121^ However, there are no reports on the isolation of secondary metabolites from *P*. *tuberculatum* leaves with herbicidal and allelopathic activity.

Our study contributed to the annotation of some allelochemicals released by *P*. *tuberculatum* leaf leaching; however, many metabolites still need to be identified in this species, considering the number of molecular networks generated in the FBMN mode. Furthermore, although LC–HRMS is a powerful tool for annotating these compounds, there are important limitations that must be considered, such as the possibility of isomers. Among the metabolites annotated, some have already been reported in the literature to be allelopathic and to have herbicidal potential, which explains the ability of *P*. *tuberculatum* leachates to inhibit germination and reduce the initial growth of *B*. *bipinnata* and *D*. *insularis*. Additional studies are necessary to understand the mechanisms of action of *P*. *tuberculatum* allelochemicals and to evaluate the selectivity of these compounds for other plant species of agronomic interest. Field tests are essential to validate their effectiveness under natural conditions and assess possible environmental and agronomic limitations.

In conclusion, the leaching pathway is used by *P*. *tuberculatum* in allelopathic interactions with other plants. The leachates demonstrated significant allelopathic inhibitory activity against the weeds *B*. *bipinnata* and *D*. *insularis*. The identification of several classes of allelochemicals, such as alkaloids, fatty acids, phenolic compounds, steroids, and terpenoids, highlights the complexity and diversity of the compounds involved in this process. To the best of our knowledge, our study is pioneering on the allelopathy of *P*. *tuberculatum* leachates. These findings provide valuable information for developing effective and sustainable weed control strategies for agricultural applications.

## Supporting Information Available

Additional tables for experimental data, analysis of variance, comparison of the sample metabolite fragmentation spectrum with the GNPS library reference spectrum, and metabolites annotated by LC–MS^E^ analysis and FBMN. Additional figures for molecular networks with LC–MS^E^ data in positive and negative ion modes via FBMN and structures of secondary metabolites annotated in *P*. *tuberculatum* leachates (PDF)

## Abbreviations Used

UPLC-ESI-QTOF-MS: liquid chromatography coupled with electrospray ionization quadrupole time-of-flight mass spectrometry
TEPB: Graziela Barroso Herbarium
G: germination percentage
GSI: germination speed index
RI: allelopathic effect response index
ANOVA: analysis of variance
LC-HRMS: liquid chromatography high-resolution mass spectrometry
PTFE: polytetrafluoroethylene
MS^E^: data-independent acquisition
GNPS: Global Natural Products Social Molecular Network
FBMN: feature-based molecular network
