# Supplementary material for: Herbicidal Activity and Metabolic Profiling of Piper tuberculatum Jacq. Leachates
Source: J Agric Food Chem. 2025 Mar 27;73(14):8160–71. doi: 10.1021/acs.jafc.4c11286 (PMC11987020; doi:10.1021/acs.jafc.4c11286)
Supplement: Supplementary file 1 — jf4c11286_si_001.pdf [file jf4c11286_si_001.pdf]

Supporting information

Herbicidal activity and metabolic profiling of *Piper tuberculatum* Jacq. leachates

Yanka Manoelly dos Santos Gaspar,[†] Álex Ap. Rosini Silva,[‡] Andreia M. Porcari,[‡] Francisca Diana da Silva Araújo[*,†]

[†] *Postgraduate Program in Agricultural Sciences, Campus Professora Cinobelina Elvas, Federal University of Piauí, Bom Jesus, PI, 64900-000, Brazil.*

[‡] *MS4Life Laboratory of Mass Spectrometry, Health Sciences Postgraduate Program, São Francisco University, Braganca Paulista, São Paulo, SP, 12916-900, Brazil.*

**Corresponding author. Email address:* diana.araujo@ufpi.edu.br

Table of Contents	S2
**Supporting Results and Discussion**	
Table S1. The percentage of seed germination (G), germination speed index (GSI), allelopathic effect response index (RI), and shoot and root lengths of *B. bipinnata* plants treated with different concentrations of *P. tuberculatum* leachates.	S3
Table S2. Percentage of seed germination (G), germination speed index (GSI), allelopathic effect response index (RI), and shoot and root lengths of *D. insularis* plants treated with different concentrations of *P. tuberculatum* leachates.	S4
Table S3. Analysis of variance of the germination percentage (G), germination speed index (GSI), allelopathic effect response index (RI), and shoot and root lengths of *B. bipinnata* plants treated with different concentrations of *P. tuberculatum* leachates.	S5
Table S4. Analysis of variance of the germination percentage (G), germination speed index (GSI), allelopathic effect response index (RI), and shoot and root lengths of *D. insularis* plants treated with different concentrations of *P. tuberculatum* leachates.	S6
Table S5. Comparison of the sample metabolite fragmentation spectrum (top spectrum) with the GNPS library reference spectrum (bottom spectrum).	S7
Table S6. Metabolites annotated by LC–MS^E analysis and molecular networking on the basis of the characteristics of *P. tuberculatum* leachates.	S14
Figure S1. Molecular networks with LC–MS^E data in positive (A) and negative (B) ion modes via feature-based molecular networks (FBMNs) obtained from *P. tuberculatum* leachates.	S15
Figure S2. Structure of secondary metabolites annotated in *P. tuberculatum* leachates. These structures are representative, and isomers are possible.	S16

Table S1. The percentage of seed germination (G), germination speed index (GSI), allelopathic effect response index (RI), and shoot and root lengths of *B. bipinnata* plants treated with different concentrations of *P. tuberculatum* leachates.

Concentration (mg of dried leaves/20 mL of 0.5% agar)	G (%)	GSI	RI	Shoot length (mm)	Root length (mm)
0	49.25	14.79	0.00	5.25	7.77
25	43.50	12.28	-0.12	3.49	4.14
50	37.25	9.56	-0.24	1.88	2.49
100	29.00	6.41	-0.41	0.74	0.83
200	15.50	2.98	-0.68	0.11	0.20

Table S2. Percentage of seed germination (G), germination speed index (GSI), allelopathic effect response index (RI), and shoot and root lengths of *D. insularis* plants treated with different concentrations of *P. tuberculatum* leachates.

**Concentration (mg of dried leaves/20 mL of 0.5% agar)**	**G (%)**	**GSI**	**RI**	**Shoot length (mm)**	**Root length (mm)**
0	46.00	20.73	0.00	2.26	5.64
25	46.75	18.77	0.01	2.45	4.61
50	39.75	14.61	-0.14	2.01	3.67
100	29.25	8.82	-0.36	1.15	1.85
200	16.75	5.25	-0.64	0.50	0.69

Table S3. Analysis of variance of the germination percentage (G), germination speed index (GSI), allelopathic effect response index (RI), and shoot and root lengths of *B. bipinnata* plants treated with different concentrations of *P. tuberculatum* leachates.

Variable	Concentration (mg of dried leaves/20 mL of 0.5% agar)	N	Mean	df	F	P
G	0	4	49.25	4	11.694	<0.001
	25	4	43.50			
	50	4	37.25			
	100	4	29.00			
	200	4	15.50			
GSI	0	4	14.79	4	17.401	<0.001
	25	4	12.28			
	50	4	9.56			
	100	4	6.41			
	200	4	2.98			
RI	0	4	0.00	4	14.383	<0.001
	25	4	-0.12			
	50	4	-0.24			
	100	4	-0.41			
	200	4	-0.68			
Shoot length	0	4	5.25	4	16.608	<0.001
	25	4	3.49			
	50	4	1.88			
	100	4	0.74			
	200	4	0.11			
Root length	0	4	7.77	4	16.083	<0.001
	25	4	4.14			
	50	4	2.49			
	100	4	0.83			
	200	4	0.20			

df: degree of freedom.

Table S4. Analysis of variance of the germination percentage (G), germination speed index (GSI), allelopathic effect response index (RI), and shoot and root lengths of *D. insularis* plants treated with different concentrations of *P. tuberculatum* leachates.

Variable	Concentration (mg of dried leaves/20 mL of 0.5% agar)	N	Mean	df	F	P
G	0	4	46.00	4	19.476	<0.001
	25	4	46.75			
	50	4	39.75			
	100	4	29.25			
	200	4	16.75			
GSI	0	4	20.73	4	17.514	0.002
	25	4	18.77			
	50	4	14.61			
	100	4	8.82			
	200	4	5.25			
RI	0	4	0.00	4	22.507	<0.001
	25	4	0.01			
	50	4	-0.14			
	100	4	-0.36			
	200	4	-0.64			
Shoot length	0	4	2.26	4	24.057	<0.001
	25	4	2.45			
	50	4	2.01			
	100	4	1.15			
	200	4	0.50			
Root length	0	4	5.64	4	34.939	<0.001
	25	4	4.61			
	50	4	3.67			
	100	4	1.85			
	200	4	0.69			

df: degree of freedom.

Table S5. Comparison of the sample metabolite fragmentation spectrum (top spectrum) with the GNPS library reference spectrum (bottom spectrum).

Alcaloids	
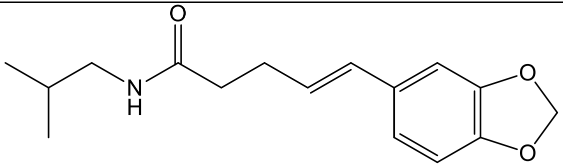	5-(1,3-benzodioxol-5-yl)-*N*-(2-methylpropyl)pent-4-enamide<br>*m/z* 276.1598 $[M+H]^+$<br>**(42)**
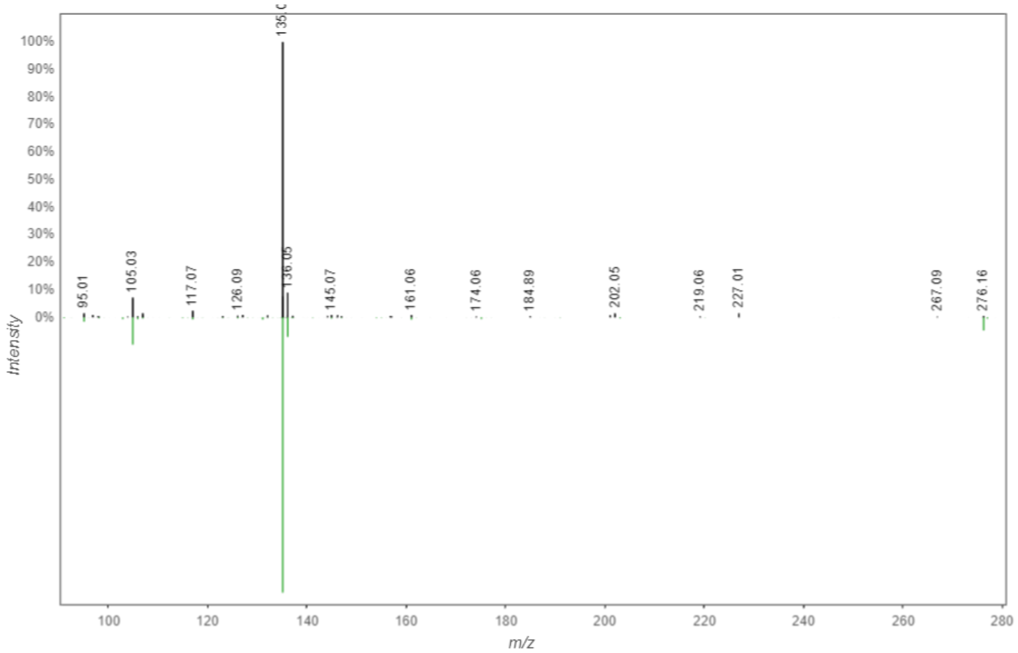	
Fatty acids	
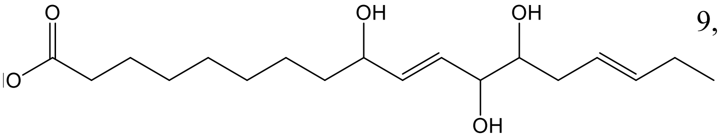	9,12,13-trihydroxy-octadec-10,15-dienoic acid<br>*m/z* 327.2171 $[M+H]^-$<br>**(43)**
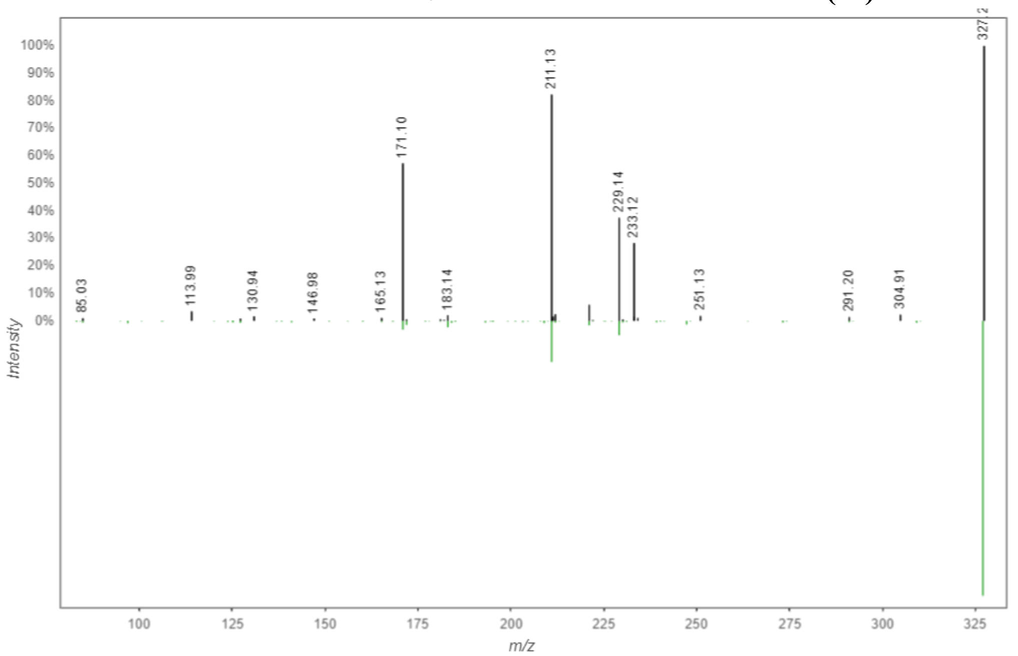	

Phenolic compounds

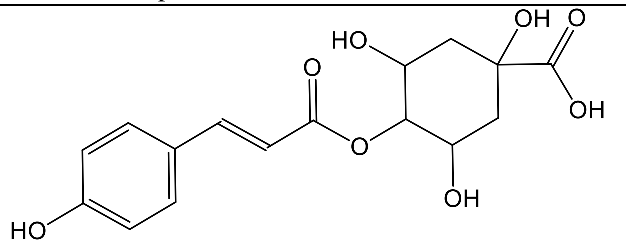

4-*p*-Coumaroylquinic acid
m/z 339.1070 [M+H]$^+$
(44)

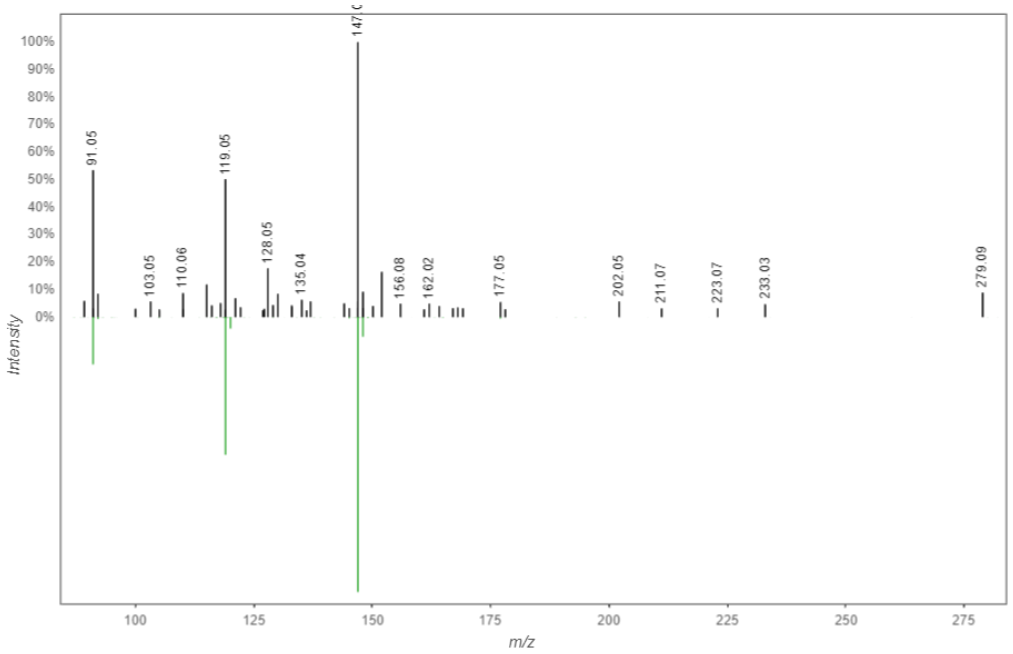

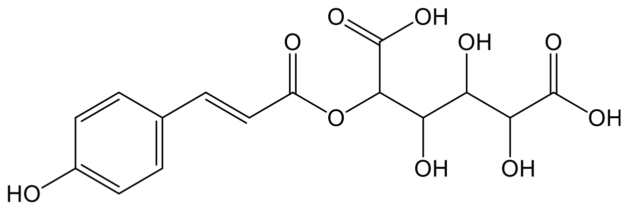

2,3,4-trihydroxy-5-[3-(4-hydroxyphenyl)prop-2-enoyl]oxyhexanedioic acid
m/z 355.0664 [M-H]$^-$
(45)

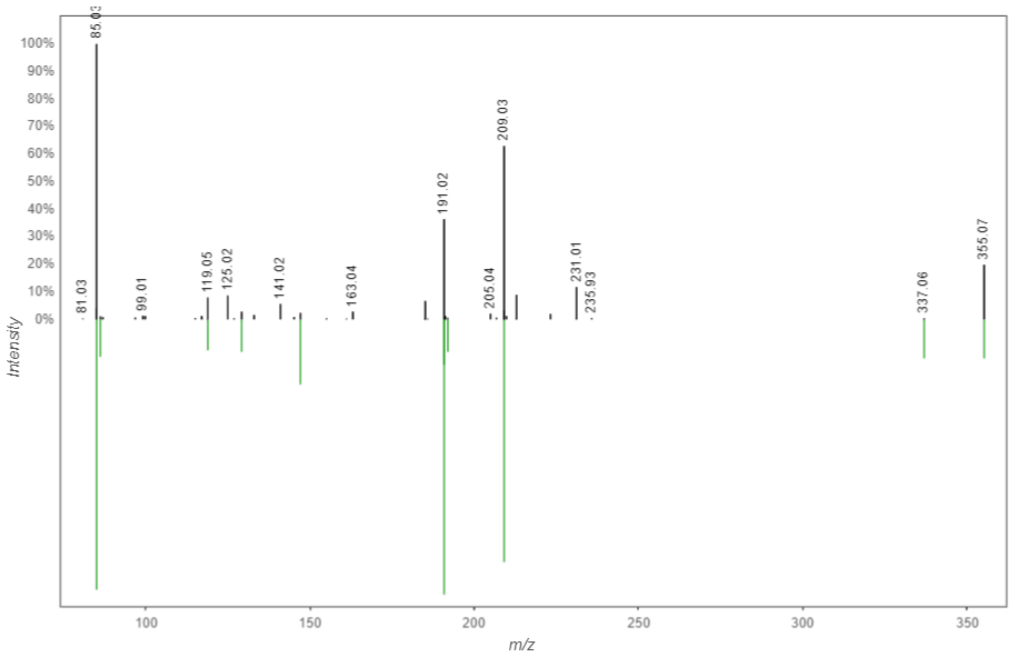

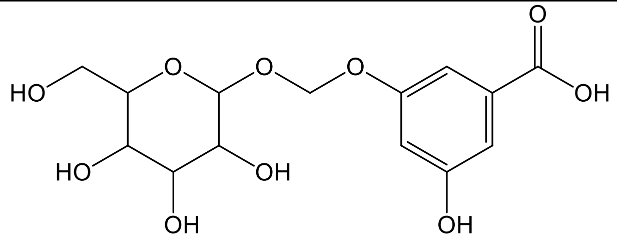

Gentisic acid 5-*O*-hexoside
m/z 315.0716 [M-H]$^-$
(46)

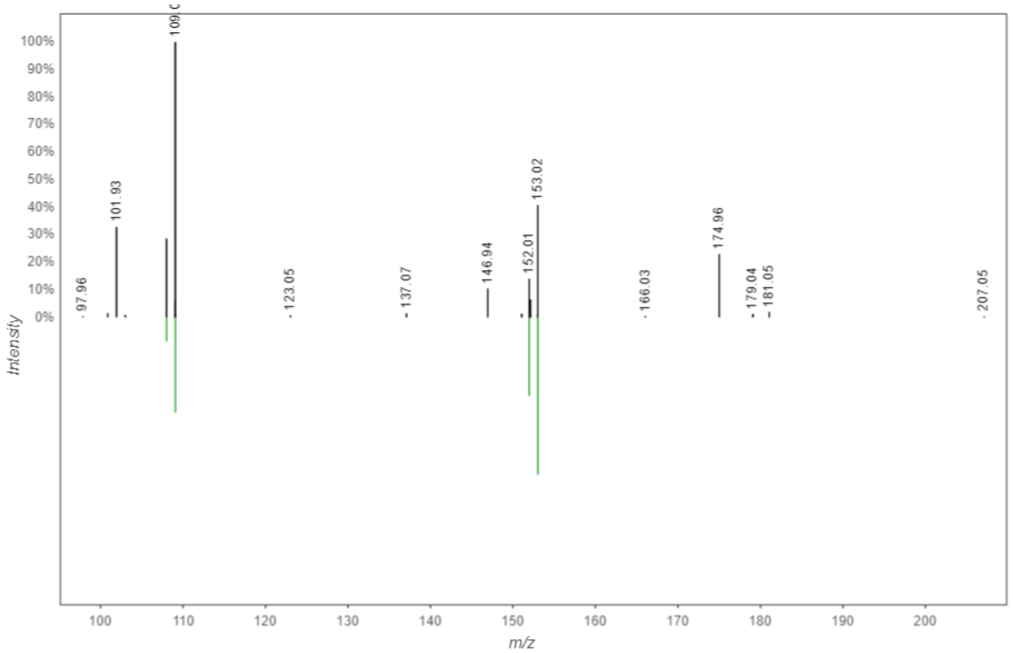

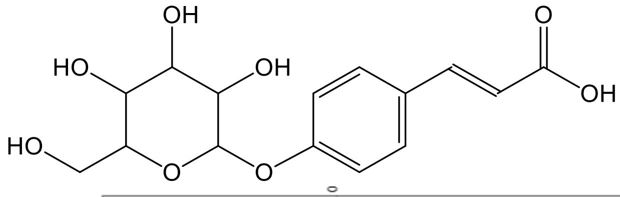

Coumaric acid 4-*O*-hexoside
m/z 325.0922 [M-H]$^-$
(47)

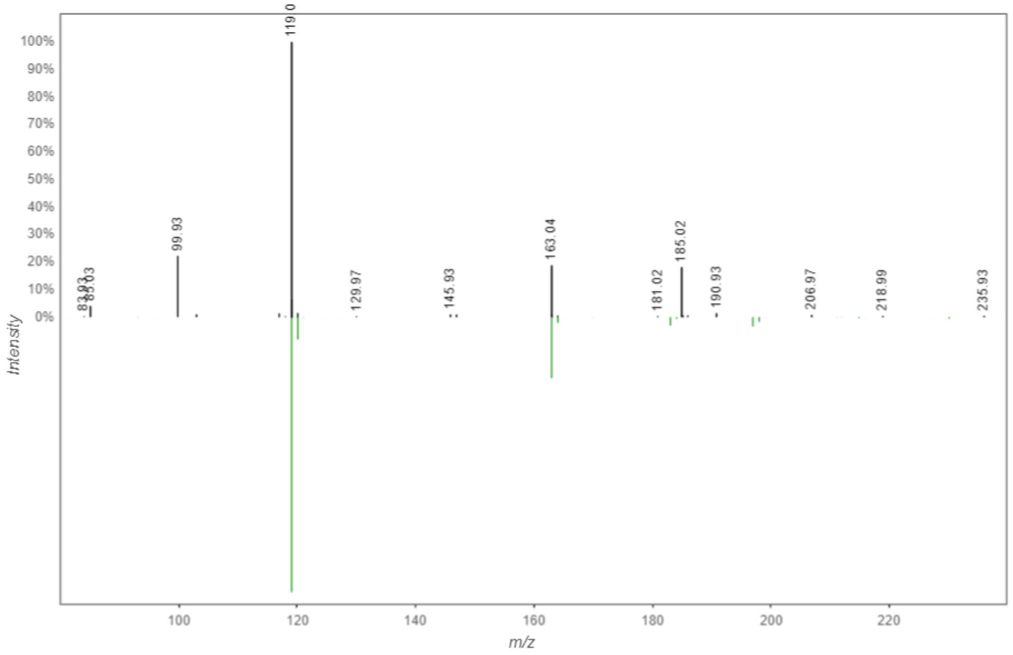

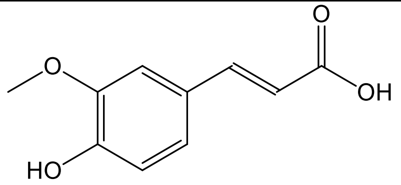

Ferulic acid
m/z 177.0550 $[M+H-H_2O]^+$
(48)

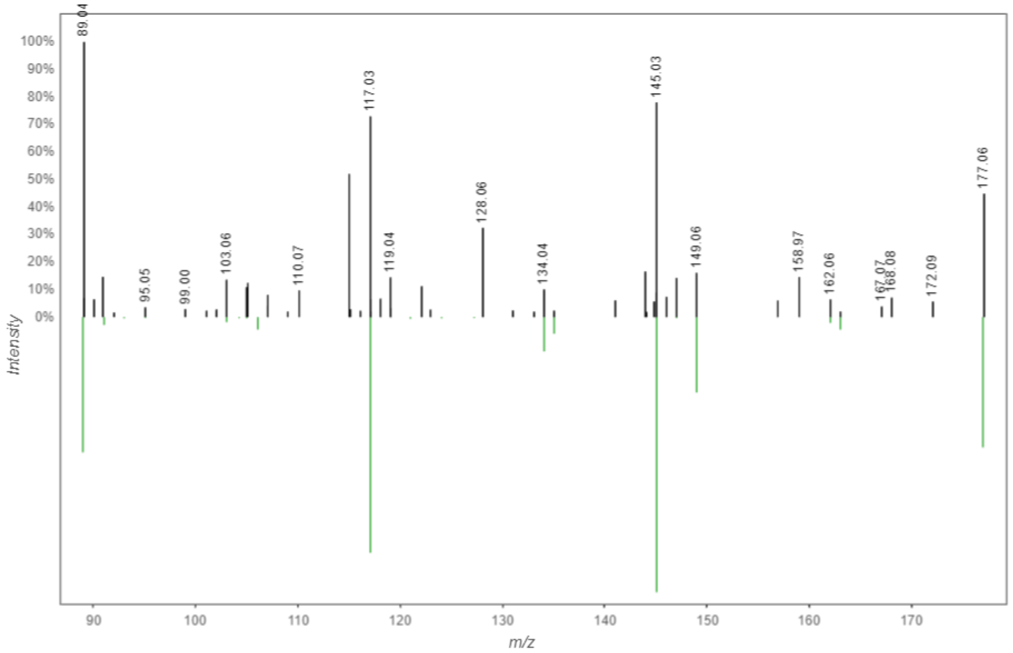

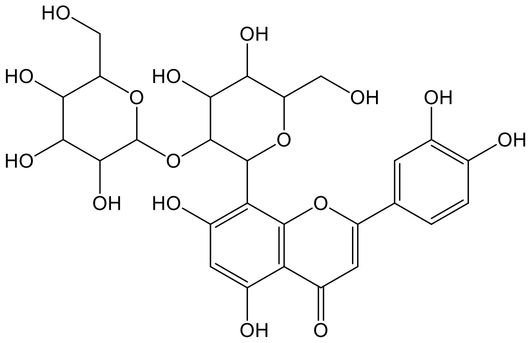

Luteoin 8-*C*-hexoside-hexoside
m/z 611.1611 $[M+H]^+$
(49)

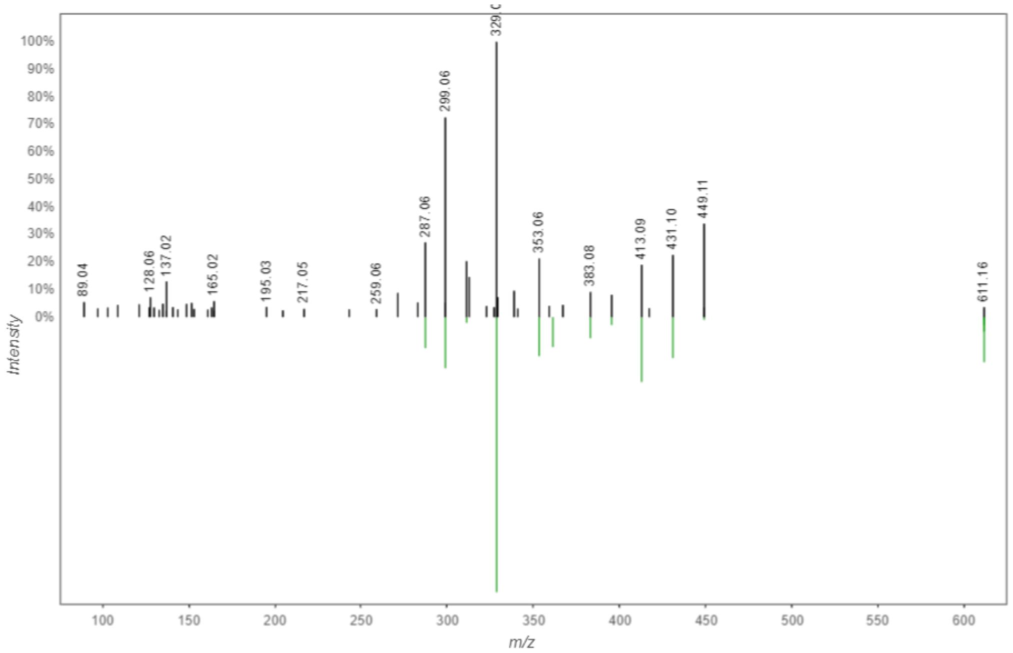

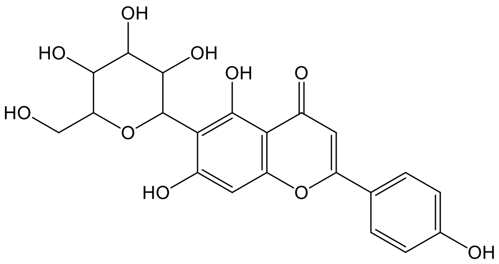

Apigenin 6-*C*-hexoside
m/z 433.1135 $[M+H]^+$
(50)

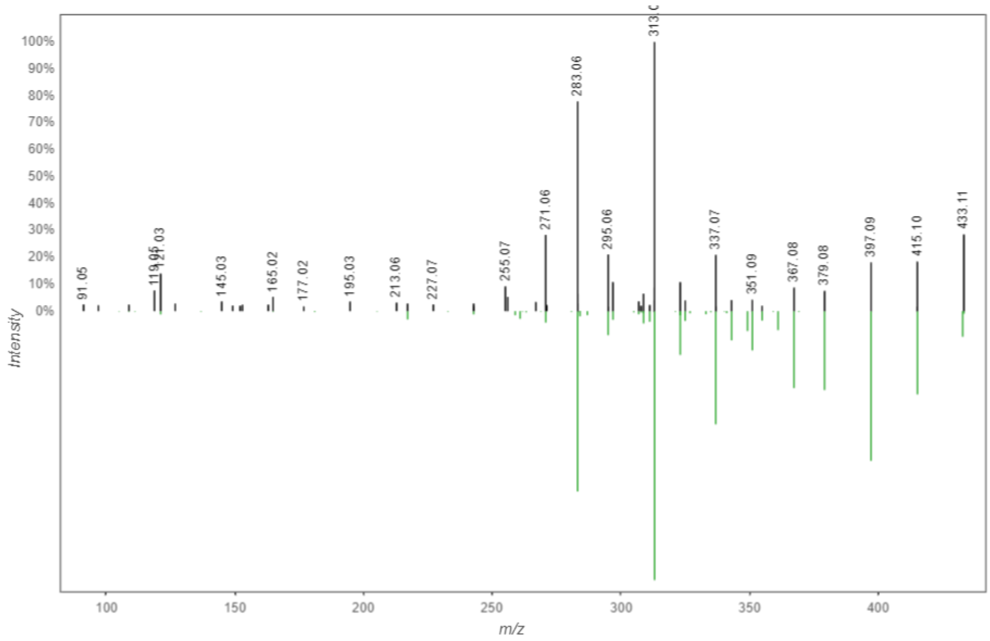

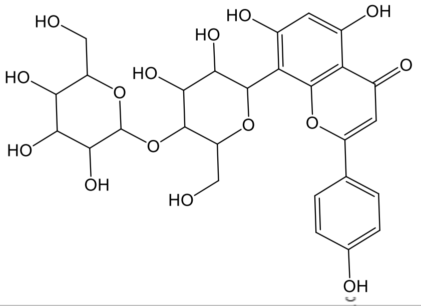

Apigenin 8-*C*-hexoside-hexoside
m/z 593.1511 $[M-H]^-$
(51)

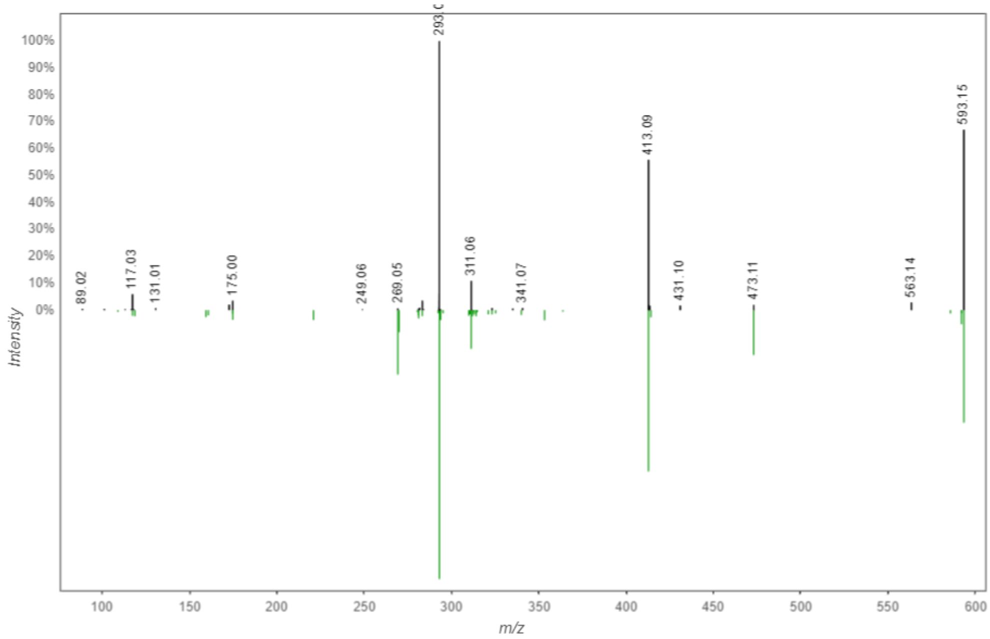

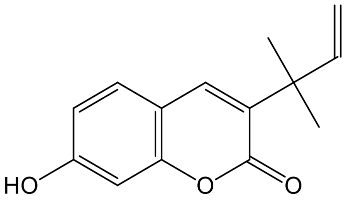

7-Hydroxy-3-(1,1-dimethylprop-2-enyl)coumarin
m/z 316.1908 $[M+NH_4]^+$
(52)

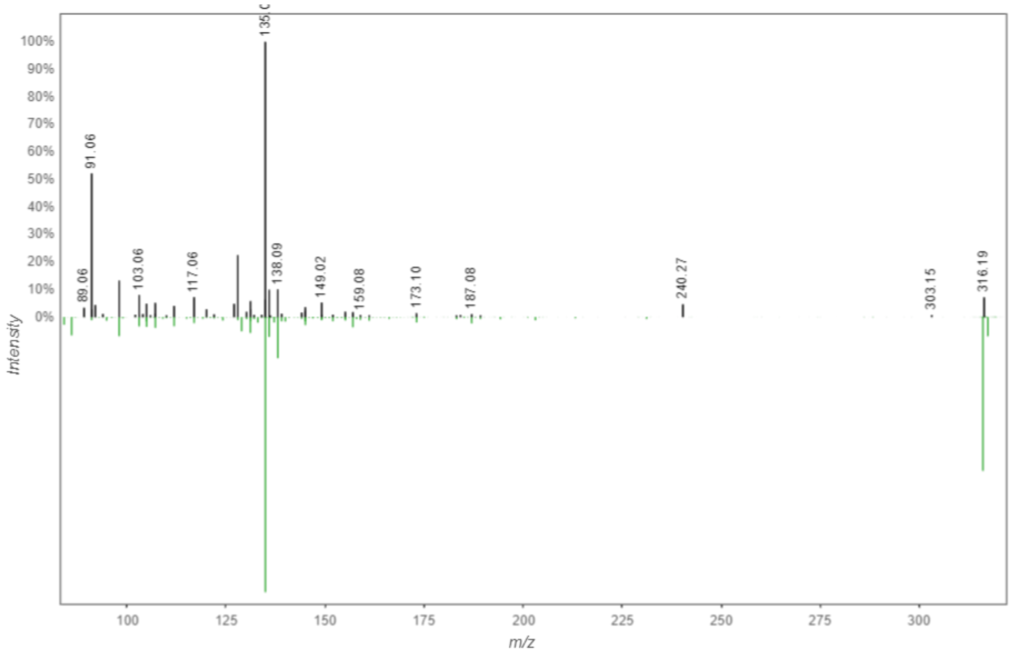

Steroids

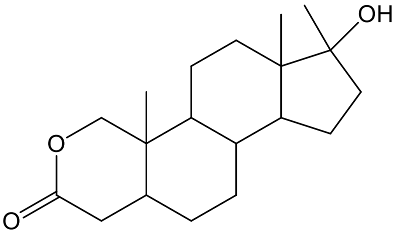

17-Epioxandrolone
m/z 307.2267 $[M+H]^+$
(53)

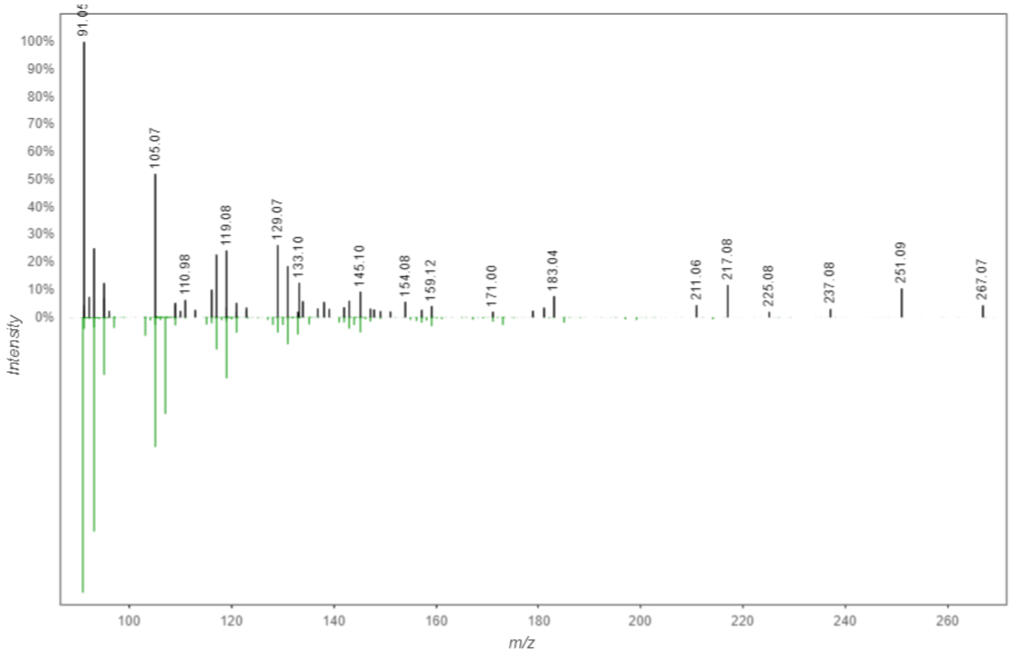

Terpenoids

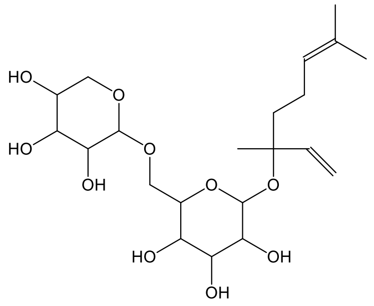

Linalool 3-*O*-hexoside-pentoside
m/z 471.2204 [M+Na]$^+$
(54)

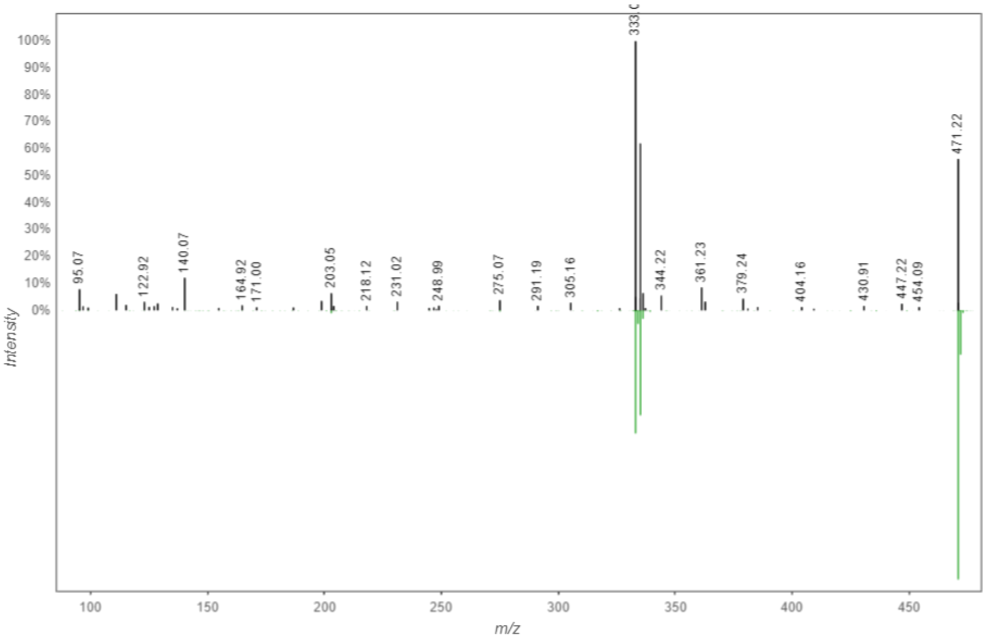

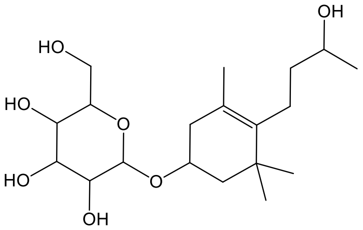

Tsangane 3-*O*-hexoside
m/z 395.2041 [M+Na-2H]$^{-}$
(55)

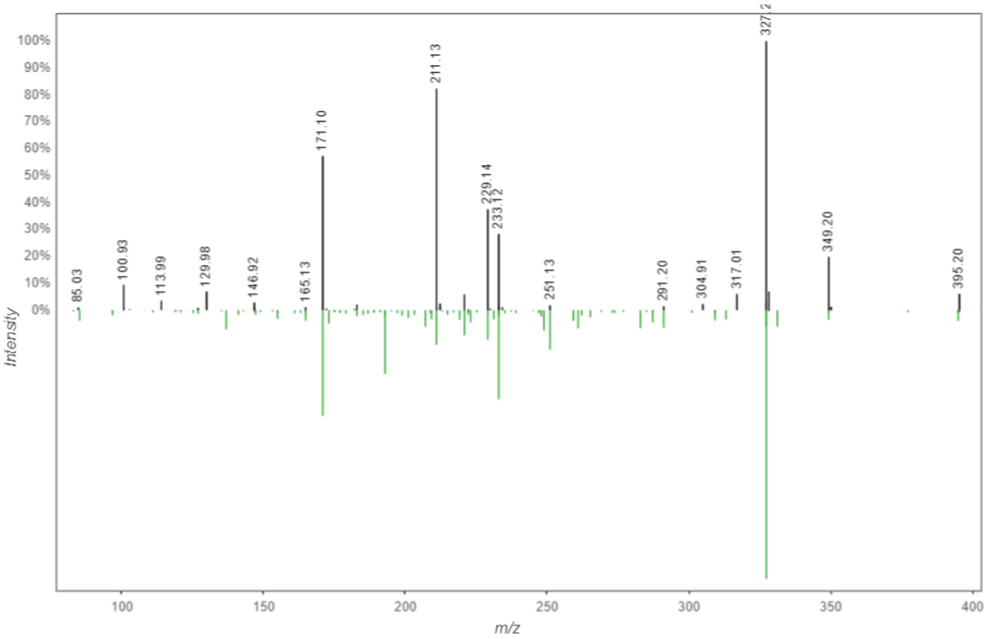

Table S6. Metabolites annotated via LC–MS^E analysis and molecular networking on the basis of the characteristics of *P. tuberculatum* leachates.

No.	Retention time (min)	Precursor ion	Adduct type	Molecular formula	Exact mass	Compound	Δ *m/z* (ppm)	Cosine score
Alkaloid								
42	14.16	276.1598	$[M+H]^+$	$C_{16}H_{21}NO_3$	275.1521	5-(1,3-Benzodioxol-5-yl)-*N*-(2-methylpropyl)pent-4-enamide	2.87	0.90
Fatty acid								
43	13.93	327.2171	$[M-H]^-$	$C_{18}H_{32}O_5$	328.2250	9,12,13-Trihydroxy-octadec-10,15-dienoic acid	3.35	0.75
Phenolic compounds								
44	6.46	339.1070	$[M+H]^+$	$C_{16}H_{18}O_8$	338.1002	4-*p*-Coumaroylquinic acid	0.00	0.83
45	6.84	355.0664	$[M-H]^-$	$C_{15}H_{16}O_{10}$	356.0743	2,3,4-Trihydroxy-5-[3-(4-hydroxyphenyl)prop-2-enoyl]oxyhexanedioic acid	3.95	0.77
46	6.91	315.0716	$[M-H]^-$	$C_{13}H_{16}O_9$	316.0794	Gentisic acid 5-*O*-hexoside	5.03	0.81
47	7.11	325.0922	$[M-H]^-$	$C_{15}H_{18}O_8$	326.1002	Coumaric acid 4-*O*-hexoside	2.44	0.80
48	8.22	177.0550	$[M+H-H_2O]^+$	$C_{10}H_{10}O_4$	194.0579	Ferulic acid	0.00	0.78
49	9.12	611.1611	$[M+H]^+$	$C_{27}H_{30}O_{16}$	610.1534	Luteoin 8-*C*-hexoside-hexoside	1.79	0.80
50	9.46	433.1135	$[M+H]^+$	$C_{21}H_{20}O_{10}$	432.1056	Apigenin 6-*C*-hexoside	8.10	0.83
51	9.49	593.1511	$[M-H]^-$	$C_{27}H_{30}O_{15}$	594.1585	Apigenin 8-*C*-hexoside-hexoside	0.20	0.80
52	16.50	316.1909	$[M+NH_4]^+$	$C_{19}H_{22}O_3$	298.1569	7-Hydroxy-3-(1,1-dimethylprop-2-enyl)coumarin	0.38	0.73
Steroids								
53	13.88	307.2267	$[M+H]^+$	$C_{20}H_{32}O_3$	320.2351	17-Epioxandrolone	0.89	0.71
Terpenoids								
54	12.88	471.2204	$[M+Na]^+$	$C_{21}H_{36}O_{10}$	448.2308	Linalool 3-*O*-hexoside-pentoside	0.84	0.80
55	13.93	395.2041	$[M+Na-2H]^-$	$C_{19}H_{34}O_7$	374.2305	Tsangane 3-*O*-hexoside	10.34	0.75

(A) (B)

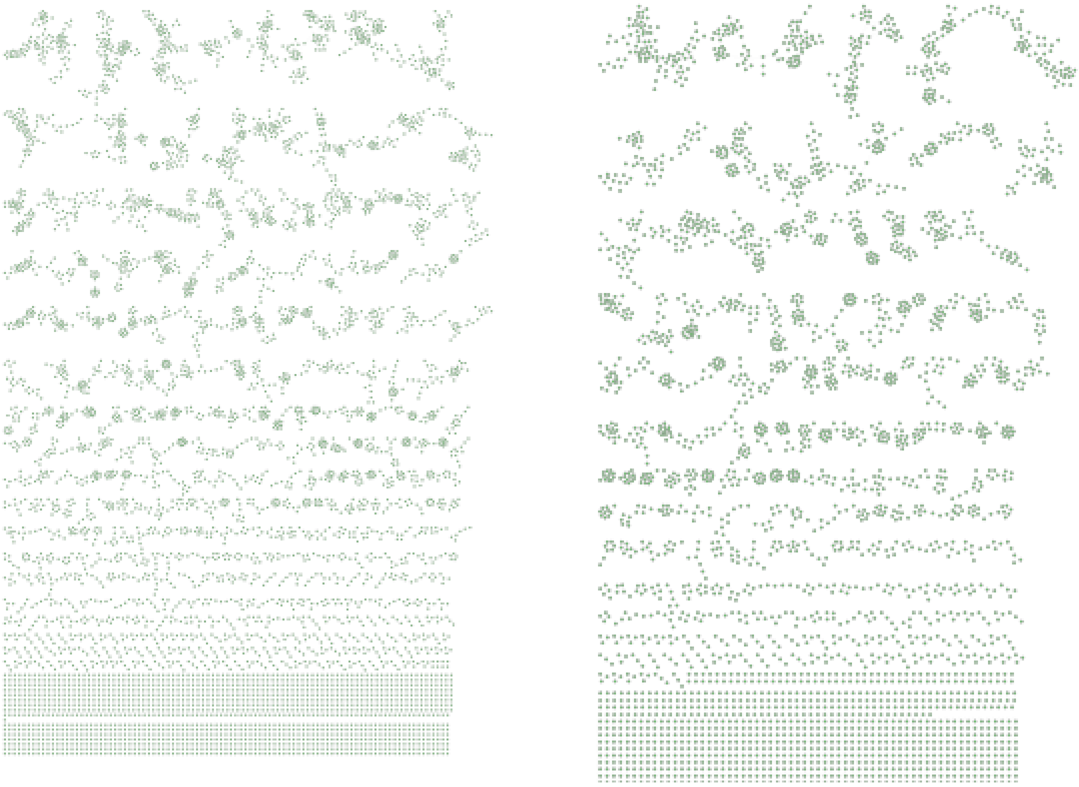

Figure S1. Molecular networks with LC–MS^E data in positive (A) and negative (B) ion modes via Feature-Based Molecular Networks (FBMNs) obtained from *P. tuberculatum* leachates.

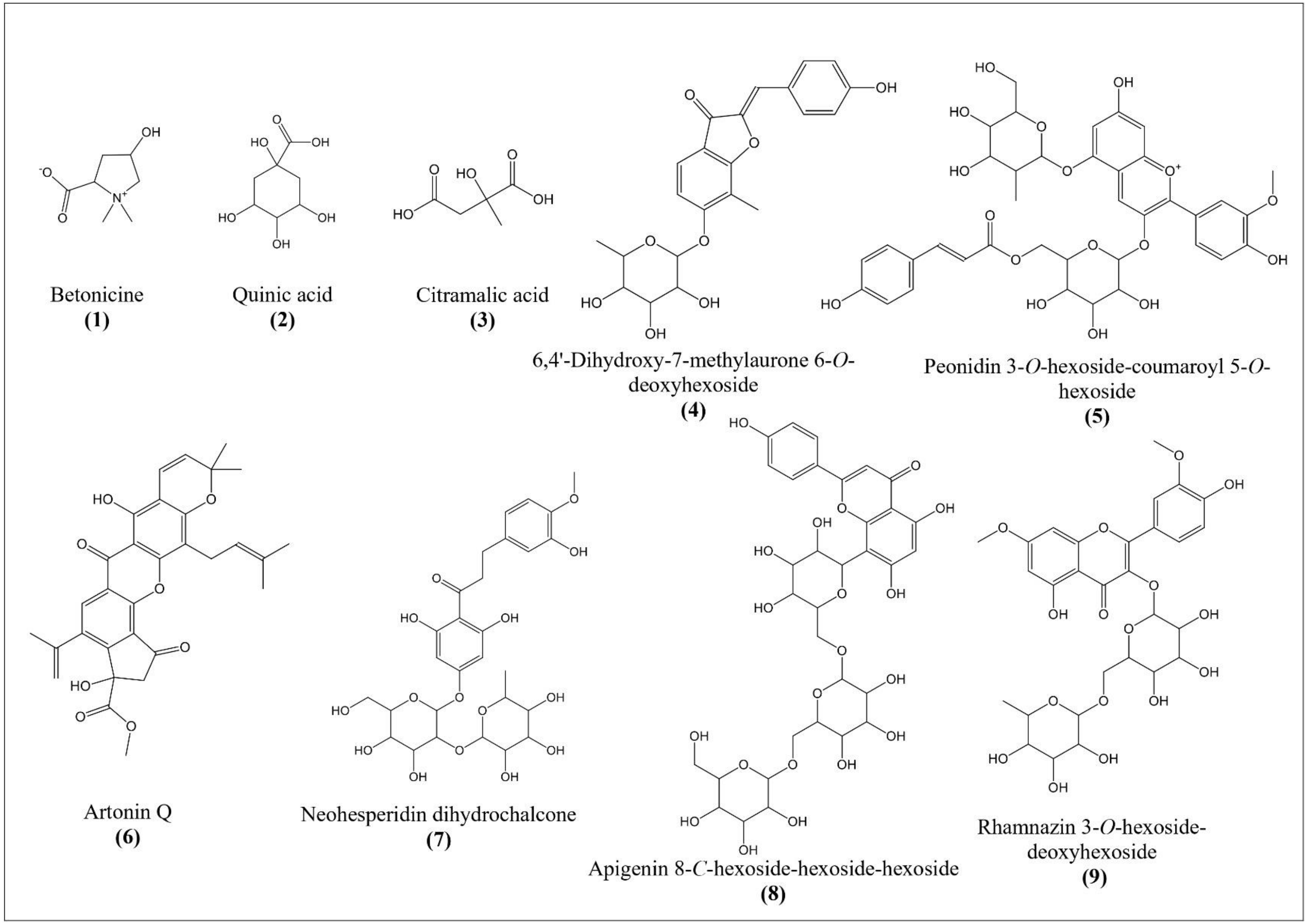

Figure S2. Secondary metabolites annotated in *P. tuberculatum* leachates. These structures are representative, and isomers are possible. To be continued…

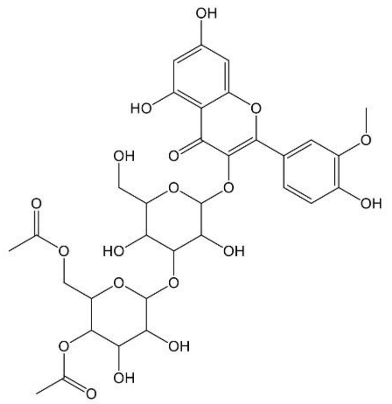

Isorhamnetin 3-*O*-hexoside-diacetyl-hexoside
(10)

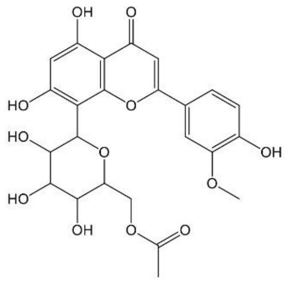

Chryseriol 8-*C*-acetyl-hexoside
(11)

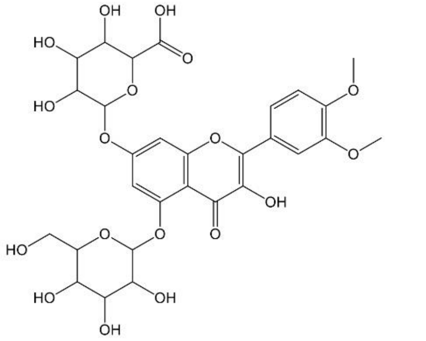

Dillenetin 5-*O*-hexoside 7-*O*-glucuronide
(12)

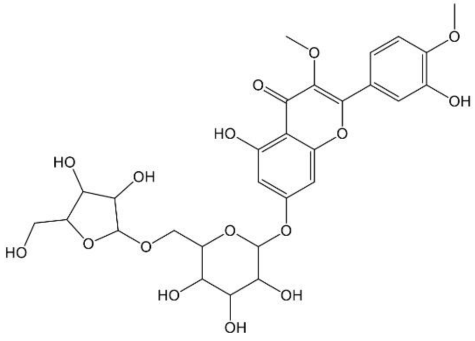

3,4'-*O*-dimethyl quercetin 7-*O*-hexoside-pentoside
(13)

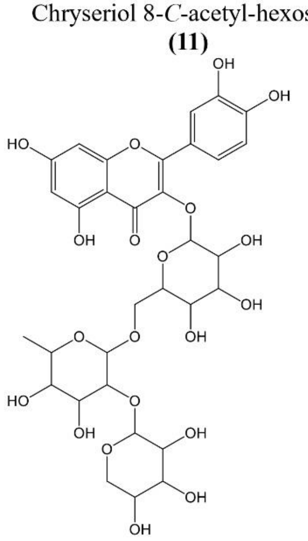

Quercetin 3-*O*-hexoside-deoxyhexoside-pentoside
(14)

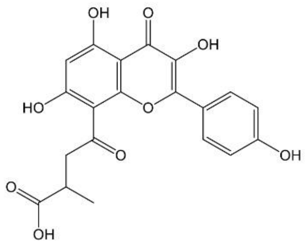

3,5,7,4'-Tetrahydroxy-8-C-(3-methylsuccinoyl)flavone
(15)

Figure S2. Secondary metabolites annotated in *P. tuberculatum* leachates. These structures are representative, and isomers are possible. To be continued…

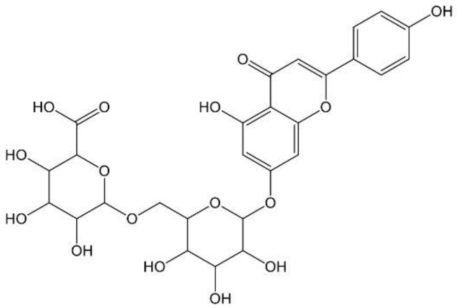

Apigenin 7-*O*-hexoside-glucuronide
(16)

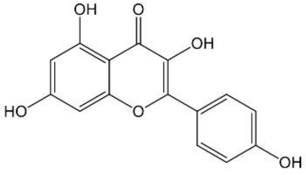

Kaempferol
(17)

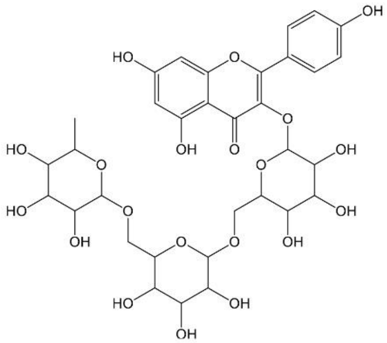

Kaempferol 3-*O*-hexoside-hexoside-deoxyhexoside
(18)

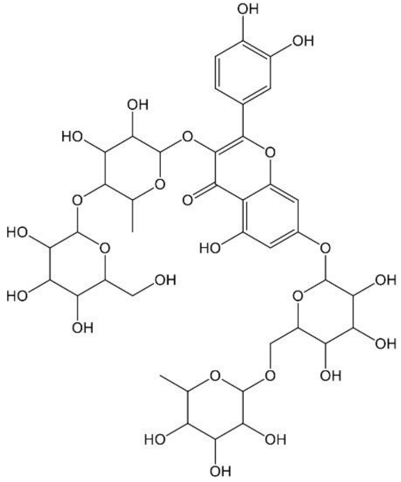

Quercetin 3-*O*-deoxyhexoside-hexoside 7-*O*-hexoside- deoxyhexoside
(19)

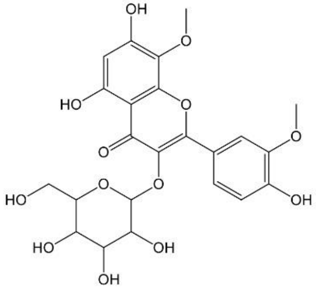

Limocitrin 3-*O*-hexoside
(20)

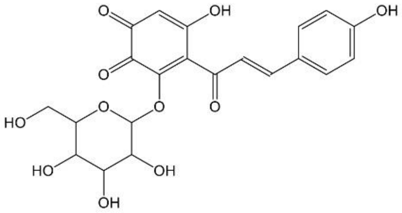

Carthamone
(21)

Figure S2. Secondary metabolites annotated in *P. tuberculatum* leachates. These structures are representative, and isomers are possible. To be continued…

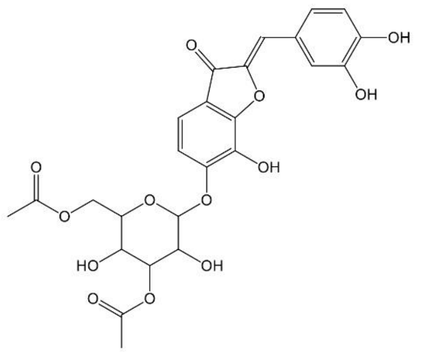

Maritimetin 6-*O*-diacetyl-hexoside
(22)

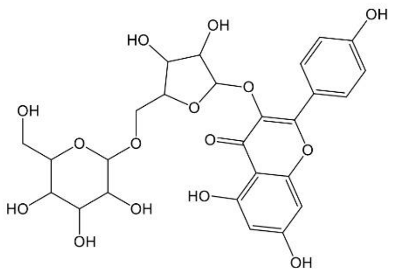

Kaempferol 3-*O*-pentoside-hexoside
(23)

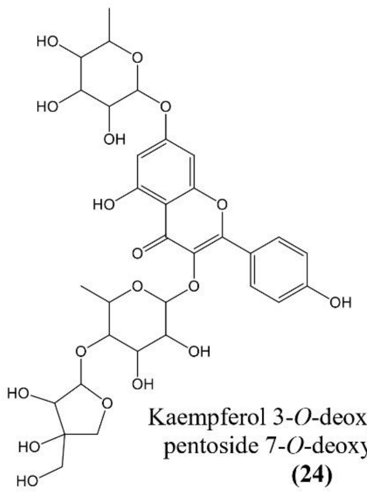

Kaempferol 3-*O*-deoxyhexoside-pentoside 7-*O*-deoxyhexoside
(24)

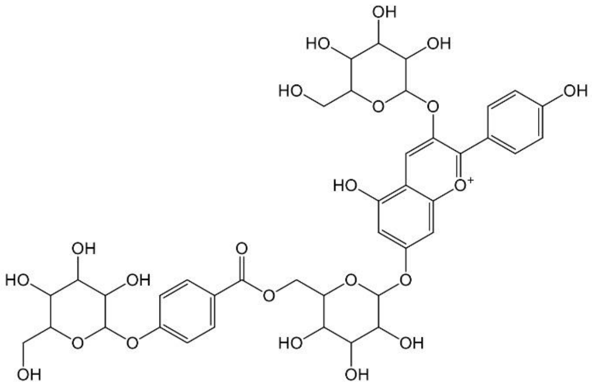

Pelargonidin 3-*O*-hexoside 7-*O*-hexoside-*p*-hydroxybenzoyl-hexoside
(25)

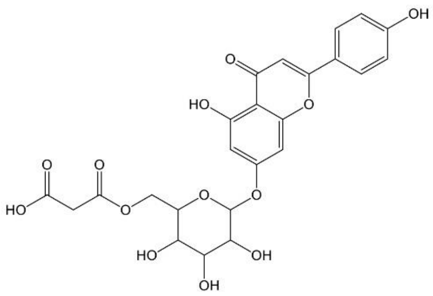

Apigenin 7-*O*-hexoside-malonyl
(26)

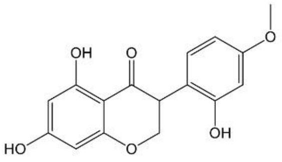

2,5,7-trihydroxy-4'-methoxyisoflavanone
(27)

Figure S2. Secondary metabolites annotated in *P. tuberculatum* leachates. These structures are representative, and isomers are possible. To be continued…

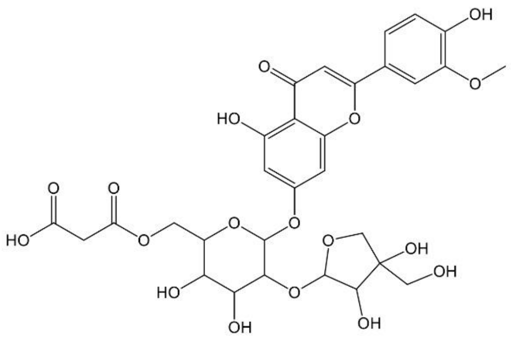

Chryseriol 7-*O*-hexoside-malonyl-pentoside
(28)

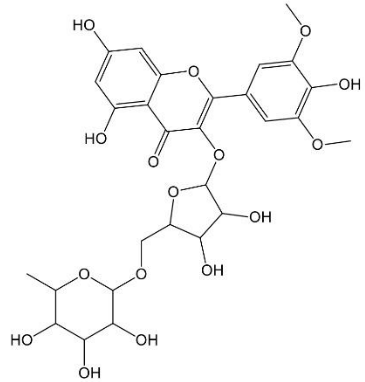

Syringetin 3-*O*-pentoside-deoxyhexoside
(29)

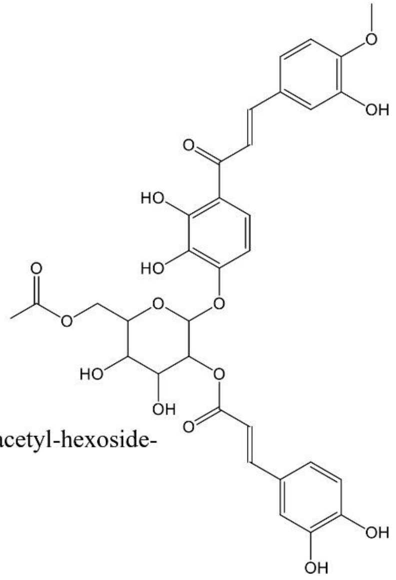

4-*O*-methyl okanin 4'-*O*-acetyl-hexoside-caffeoyl
(30)

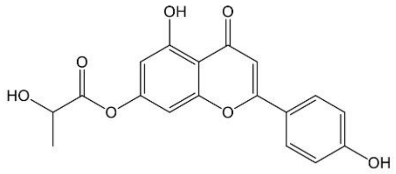

Apigenin 7-*O*-lactate
(31)

Figure S2. Secondary metabolites annotated in *P. tuberculatum* leachates. These structures are representative, and isomers are possible. To be continued…

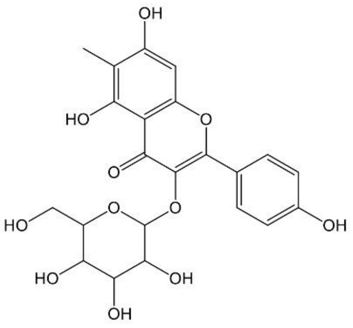

6-*C*-Methylkaempferol 3-*O*-hexoside
(32)

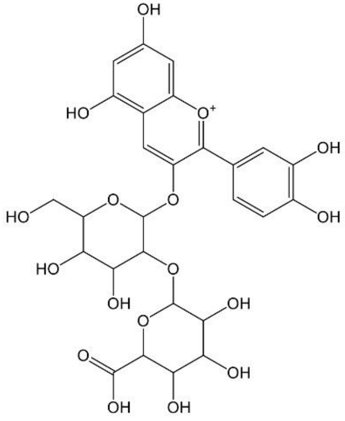

Cyanidin 3-*O*-hexoside-glucuronide
(33)

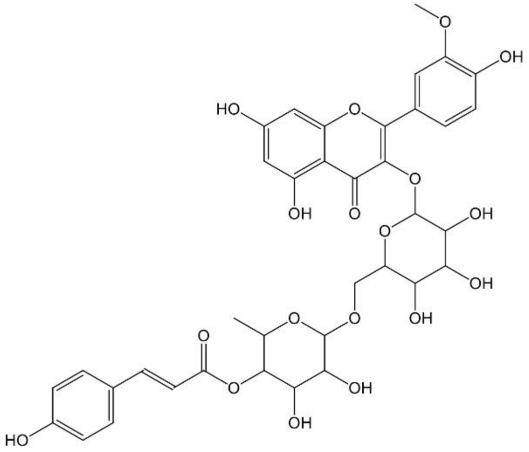

Isorhamnetin 3-*O*-hexoside-deoxyhexoside-coumaroyl
(34)

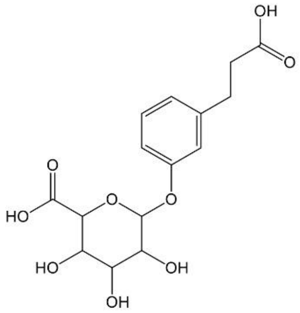

3-(4-Hydroxyphenyl)propanoic acid 3-*O*-glucuronide
(35)

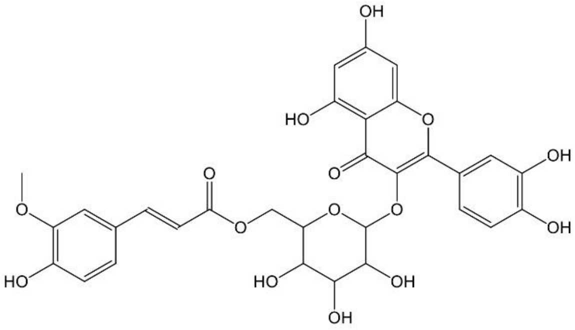

Quercetin 3-*O*-hexoside-feruloyl
(36)

Figure S2. Secondary metabolites annotated in *P. tuberculatum* leachates. These structures are representative, and isomers are possible. To be continued…

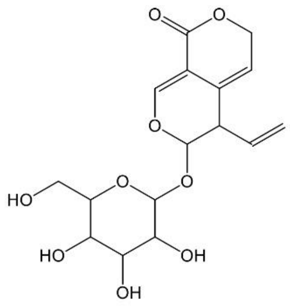

Gentiopicroside
(37)

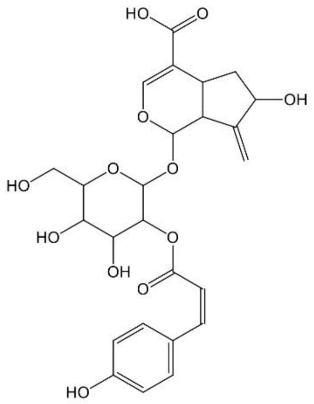

2'-*O*-coumaroylgardoside
(38)

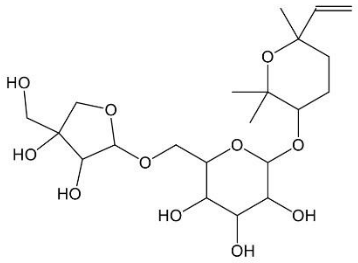

Linalool oxide D 3-*O*-hexoside-pentoside
(39)

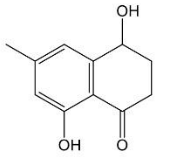

Shinanolone
(40)

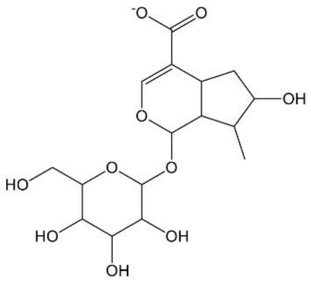

Loganate
(41)

Figure S2. Secondary metabolites annotated in *P. tuberculatum* leachates. These structures are representative, and isomers are possible.
